# Genomic Response to Vitamin D Supplementation in the Setting of a Randomized, Placebo-Controlled Trial

**DOI:** 10.1016/j.ebiom.2018.04.010

**Published:** 2018-04-10

**Authors:** Antonio J. Berlanga-Taylor, Katharine Plant, Andrew Dahl, Evelyn Lau, Michael Hill, David Sims, Andreas Heger, Jonathan Emberson, Jane Armitage, Robert Clarke, Julian C. Knight

**Affiliations:** a Computational Genomics Analysis and Training (CGAT), Weatherall Institute of Molecular Medicine, John Radcliffe Hospital, University of Oxford, Oxford OX3 9DS, UK; b Wellcome Centre for Human Genetics (WHG), Nuffield Department of Medicine, University of Oxford, Roosevelt Drive, Oxford OX3 7BN, UK; c MRC-PHE Centre for Environment and Health, Department of Epidemiology & Biostatistics, School of Public Health, Faculty of Medicine, Imperial College London, St Mary's Campus, Norfolk Place, London W2 1PG, UK; d Clinical Trial Service Unit and Epidemiological Studies Unit, Nuffield Department of Population Health, University of Oxford, Roosevelt Drive, Oxford OX3 7LF, UK; e MRC Population Health Research Unit, Nuffield Department of Population Health, University of Oxford, Roosevelt Drive, Oxford OX3 7LF, UK

**Keywords:** Vitamin D, Functional genomics, Clinical trial, Quantitative trait locus

## Abstract

**Background:**

Vitamin D deficiency has been associated with multiple diseases, but the causal relevance and underlying processes are not fully understood. Elucidating the mechanisms of action of drug treatments in humans is challenging, but application of functional genomic approaches in randomized trials may afford an opportunity to systematically assess molecular responses.

**Methods:**

In the Biochemical Efficacy and Safety Trial of Vitamin D (BEST-D), a double-blind, placebo-controlled, dose-finding, randomized clinical trial, 305 community-dwelling individuals aged over 65 years were randomly allocated to treatment with vitamin D_3_ 4000 IU, 2000 IU or placebo daily for 12 months. Genome-wide genotypes at baseline, and transcriptome and plasma levels of cytokines (IFN-γ, IL-10, IL-8, IL-6 and TNF-α) at baseline and after 12 months, were measured. The trial had >90% power to detect 1.2-fold changes in gene expression.

**Findings:**

Allocation to vitamin D for 12-months was associated with 2-fold higher plasma levels of 25-hydroxy-vitamin D (25[OH]D, 4000 IU regimen), but had no significant effect on whole-blood gene expression (FDR < 5%) or on plasma levels of cytokines compared with placebo. In pre-specified analysis, rs7041 (intron variant, *GC*) had a significant effect on circulating levels of 25(OH)D in the low dose, but not in the placebo or high dose vitamin D regimen. A gene expression quantitative trait locus analysis (eQTL) demonstrated evidence of 31,568 *cis*-eQTLs (unique SNP-probe pairs) among individuals at baseline and 34,254 after supplementation for 12 months (any dose). No significant associations involving vitamin D supplementation response eQTLs were found.

**Interpretation:**

We performed a comprehensive functional genomics and molecular analysis of vitamin D supplementation in a randomized, placebo-controlled trial. Although this study was limited to mostly Caucasian individuals aged over 65 years, the results differ from many previous studies and do not support a strong effect of vitamin D on long-term transcriptomic changes in blood or on plasma cytokine levels. The trial demonstrates the feasibility of applying functional genomic and genetic approaches in randomized trials to assess molecular and individual level responses.

**Key Result:**

Supplementation with high-dose vitamin D in older people for 12 months in a randomized, placebo-controlled trial had no significant effect on gene expression or on plasma concentrations of selected cytokines.

**Trial Registration:**

SRCTN registry (Number 07034656) and the European Clinical Trials Database (EudraCT Number 2011-005763-24).

## Highlights

• We carried out molecular analyses in a randomized, placebo-controlled, clinical trial of daily vitamin D supplementation.
• Participants were Caucasian, aged >65 years with measurements at baseline and 12 months for placebo, 2000 IU or 4000 IU.
• We did not find significant differences in genomic, transcriptomic or cytokines in blood despite large changes in 25(OH)D levels.

Many studies have reported associations of vitamin D deficiency with different diseases, but little is known about the causal relevance of such associations. Genomics technologies can provide important tools for discovery and validation of disease and drug mechanisms in clinical trials, but have not been widely used. Vitamin D is known to have genome-wide effects, but results have mainly come from laboratory models or small studies in humans. Results of previous studies differ, possibly reflecting the complexity of synthesis and metabolism of vitamin D. In the present study, we used a randomized placebo-controlled trial, but did not observe any significant differences in expression in genes (genome-wide) or on plasma concentrations of selected cytokines in community-dwelling older individuals. Vitamin D is essential for human physiology and undoubtedly has multiple effects. Such effects are likely to vary between cells, tissues, individuals, populations and environmental circumstances. The mechanisms which regulate vitamin D are complex and likely to be robust to such differences. The present study shows that integrating high throughput molecular measurements is feasible in a community-based clinical trial and the results will inform future research investigating the mechanisms of action of vitamin D.

## 1 Introduction

Randomized controlled trials afford reliable approaches to understand the causal relevance of drug treatments and can also help our understanding of disease mechanisms by relating changes in biomarkers with incidence of disease or with surrogate markers of disease. Advances in molecular methods now permit use of high-throughput functional genomics strategies in clinical trials. Application of such approaches has been under-utilized to date, with previous studies focusing on comparisons of transcriptomes to understand mechanisms and identify novel biomarkers (Beck et al., 2014). Animal and experimental models of disease pathogenesis have limited ability for translation into humans, particularly in the context of complex diseases (Seok et al., 2013, Takao and Miyakawa, 2015), while incomplete knowledge of mechanisms contributes to inconclusive findings in randomized trials and the current high failure rate in late stage drug development. Hence, there is an urgent need to demonstrate the value of combining functional genomic approaches, including genome-wide genotyping and gene expression profiling, together with measurements of biochemical and clinical markers in clinical trials to enhance our understanding of the pathophysiological processes and mechanisms of action of novel drug treatments. Such approaches may also allow high-throughput assessment of cellular and molecular responses at group and individual levels that can also be integrated with effects on clinical outcomes data. Hence, integrated analysis may yield clinically relevant insights about treatment that could guide the design of large outcome trials.

This study applied functional genomics methods to investigate the molecular response to vitamin D supplementation. In addition to the established role of vitamin D in calcium metabolism and bone disease, accumulating evidence suggests a possible role of vitamin D in immune function and inflammatory diseases (Bouillon et al., 2008). Previous studies have investigated the associations of vitamin D with gene expression (Carlberg et al., 2013, Hossein-Nezhad et al., 2013, Ramagopalan et al., 2010), but these have typically been cross-sectional, in experimental models, involved relatively small sample sizes or lacked placebo controls. Moreover, no previous studies have assessed the impact of genome-wide genetic variation on responses to vitamin D supplementation.

The aim of the work described here was to examine the molecular responses to vitamin D supplementation in a randomized, placebo-controlled trial. To achieve this, we investigated changes in response to treatment after 12 months in whole blood transcriptomes and plasma levels of cytokines, in addition to genetic determinants of individual responses on circulating 25-hydroxy vitamin D (25[OH]D) and genome-wide gene expression, by comparing a total of 305 individuals allocated to daily treatment with vitamin D at either 4000 IU, 2000 IU or placebo in the BEST-D trial (Hin et al., 2017).

## 2 Methods

Details of the design, baseline characteristics and data analysis plan and results of the BEST-D trial have been reported previously (Clarke et al., 2015, Hin et al., 2017). Briefly, the primary objectives of BEST-D were to compare the effects on plasma levels of 25(OH)D and to determine the proportion of participants with plasma 25(OH)D levels > 90 nmol/L after one year of supplementation with 4000 IU or 2000 IU of vitamin D_3_ versus placebo. BEST-D was designed as an intention-to-treat, double-blind, placebo-controlled, dose-finding, randomized clinical trial. Written informed consent was provided by all participants. BEST-D was approved by the National Research Ethics Service (NRES) Committee South Central–Oxford B, the Thames Valley Primary Care Research Partnership, a Clinical Trial Authorization from MHRA, and is included on the National Institute for Health Research (NIHR) Trial portfolio.

Eligible participants were ≥65 years of age, living in the community and ambulatory. Participants were randomized in a ratio of 1:1:1 to each group using a minimization algorithm balanced for age group (65–69, 70–74, ≥75 years), gender, body mass index (BMI), smoking history, ethnicity and history of fracture. Of 1122 individuals who were invited to participate, 313 (33%) agreed to receive a visit from a study nurse for randomization and 305 (32%) were successfully randomized between 24 September 2012 and 14 March 2013. All data and results were handled according to the trial and institutional guidelines in secure servers within the University of Oxford. All comparisons were conducted by intention-to-treat analyses using a pre-specified plan for the molecular data analysis (Supplementary information).

### 2.1 Procedures

Briefly, a research nurse visited participants at their homes to obtain medical history, samples and measurements. PAXgene Blood RNA tubes (Qiagen) were used to ensure RNA stability without needing immediate processing. Biological samples were transported at 2–4 °C and then stored at −80 °C. RNA and DNA samples were processed at the end of the study as detailed below. Plasma 25(OH)D levels were measured using an Access 2 immunoassay analyzer (Beckman Coulter Ltd., High Wycombe, England) complying with the quality assurance DEQAS scheme. Further details have been described previously (Hin et al., 2017). Plasma cytokines were measured using a MesoScale Discovery multi-spot assay system. The V-Plex pro-inflammatory panel 1 kit was used, with detection antibodies for interferon-gamma (IFN-γ), interleukin 6 (IL-6), interleukin 8 (IL-8), interleukin 10 (IL-10) and tumor necrosis factor alpha (TNF-α). In brief, assays were sandwich electrochemiluminescence (ECL) immunoassays. Between-run precision over the experiment for IFN-γ was 11.39% at 91.78 pg/mL and 10.73% at 10.73 pg/mL; for IL-6 11.15% at 49.20 pg/mL and 13.02% at 8.85 pg/mL; for IL-8 9.38% at 32.67 pg/mL and 12.76% at 6.75 pg/mL; for IL-10 13.60% at 22.01 pg/mL and 13.82% at 4.49 pg/mL; and for TNF-α 12.29% at 11.15 pg/mL and 14.36% at 3.19 pg/mL.

#### 2.1.1 DNA Extraction and Genotyping

Genomic DNA was extracted from the buffy coat layer using DNeasy Blood and Tissue Kit (Qiagen), and quantified by NanoDrop (Thermo Fisher Scientific; Waltham, MA) and Agilent 2100 Bioanalyzer (Agilent Technologies). 299 samples (of 305 possible) were available for DNA isolation and were processed over a single batch. Genotyping was performed using the Illumina Infinium HumanOmniExpress-24v1-0 (Illumina) beadchips following the Infinium HTS protocol (Illumina) at the Oxford Genomics Centre (WHG). Sample concentration was measured using PicoGreen (Thermo Fisher Scientific) and normalized. In total, 716,503 single nucleotide polymorphisms (SNPs) were genotyped. The genotype call rate cut-off was <98%. The overall call rate was 99.75% with one sample removed at this stage (genotype call rate 96.8%).

#### 2.1.2 RNA Extraction, cDNA Conversion and Microarray Measurements

Total RNA was isolated from whole blood samples using PAXgene Blood RNA Kit (Qiagen) with recovery of RNA populations of <200 nucleotides and globin messenger RNA clearance using the GLOBINclear Kit (Ambion). Quantification of RNA and quality measures were assessed using Agilent 2100 Bioanalyzer (Agilent Technologies) and Nanodrop (Thermo Fisher Scientific). Complementary DNA synthesis, labelling and microarray hybridization were performed at the Oxford Genomics Centre using Illumina Human-HT-12v4 Expression BeadChip (Illumina). In total, 574 samples were available for further processing after microarray measurements (of 610 possible from the 305 participants who completed the study). Thirty samples were not available at end of study, four samples failed RNA quality metrics before array hybridization and two during array processing. Three additional samples were identified as of low quality due to low amounts of complementary RNA upon further inspection. Arrays were run in batches of 96 randomized samples.

### 2.2 Statistical Analysis

#### 2.2.1 A Priori Power for Detecting Gene Expression Differences

Statistical power calculations for gene expression analysis for in vivo studies are not well documented. We estimated statistical power using available data from vitamin D treated human cell line experiments (Ramagopalan et al., 2010, Wang et al., 2005). We estimated that vitamin D_3_ supplementation may alter gene expression by 1.5 to 3-fold differences. Sample size was prioritized for the main trial outcomes (circulating levels of 25[OH]D). With a fixed sample size of 100 per group for the BEST-D trial, taking a two-sided alpha of 0.05 for a two-sample *t*-test, beta of 10%, standard deviation of 0.7, and equal size group we estimated we would be able to detect effect size differences in mean gene expression values of 1.25-fold change per gene. For paired tests, 1.17-fold changes would be detectable at the same beta. These estimates assume single gene tests with calculations performed using the base package in R and pwr (v1.1–3). Calculations using the R package sizepower (v1.48.0), which is specific for microarray experiments, for matched samples with the following assumptions: mean number of false positives = 1, genes not expected to be differentially expressed = 10,000, mean difference in log-expression between comparison groups = 1.20, standard deviation = 0.7, and sample size = 100, indicated we had complete power to detect fold changes of 1.2.

#### 2.2.2 Genome-wide SNP Processing and Statistical Analysis

Quality control was carried out using standard approaches (Anderson et al., 2010, Ritchie et al., 2015) and included assessment of gender miss-identification; subject relatedness, duplication and divergent ancestry; individuals with elevated missing data rates or outlying heterozygosity rate; identification of markers (SNPs) with excessive missing data rates; identification of differing genotype call rates between groups; SNP quality (filtering of monomorphic SNPs; SNPs with missing values or nonsense values; low call rate; violation of Hardy-Weinberg equilibrium; duplication; and minimum allele frequency). We removed low quality markers followed by individuals. We excluded non-autosomal variants. We used the following criteria for filtering: call rates > 98%; minor allele frequency (MAF) > 10%; Hardy-Weinberg equilibrium threshold of 1 × 10^−6^. This resulted in 19 SNPs with a significantly different (*p*-value <.01) missing data rate between cases and controls (treated vs placebo) being excluded; 4893 variants due to missing genotype data; 20 variants due to Hardy-Weinberg equilibrium and 193,723 with MAF < 10%. In total, 497,136 variants passed QC filters. Of 299 genotyped individuals, eleven were excluded after QC: one individual was excluded due to low genotype call rate (<98%); two due to gender misidentification; two due to relatedness (identity-by-descent value > 0.1875); three due to ancestry other than Caucasian, and three due to a high genotype failure rate (≥0.03) and/or a heterozygosity rate ± 3 SD from the mean (Supplementary Fig. 1).

Linear regression association tests were conducted using frequentist methods with PLINK version 1.90 (Purcell et al., 2007). We corrected for baseline vitamin D circulating levels, vitamin D intake (assessed at baseline), season (based on date of trial recruitment), gender, age, baseline BMI, medical history (incident fracture, incident respiratory infection, diabetes, heart disease, chronic obstructive pulmonary disease, asthma) and current smoking status. To explore genetic determinants of 25(OH)D levels we only considered SNPs previously identified by GWAS (Wang et al., 2010) and which were included in the genotyping array (rs12794714 [*CYP2R1*], rs2282679 [*GC*], rs7041 [*GC*] and rs7944926 [*DHCR7/NADSYN1*]). We utilized an adaptive Monte Carlo permutation as implemented by PLINK to derive empirically determined significance values.

#### 2.2.3 Quality Control, Normalization of Microarray Data and Differential Gene Expression Analysis

Quality assessment of gene expression data included visual analysis of un-normalized data; analysis of built-in control probes; sample outlier detection and estimation of the proportion of probes expressed across samples (Shi et al., 2010a) (Supplementary Fig. 2). Outlier detection was carried out using arrayQualityMetrics v3.24.0 (Kauffmann et al., 2009). We removed samples that failed three criteria based on the package's internal scores of individual array quality, homogeneity between arrays and between array comparisons. We found that 11 samples were classed as outliers by all three methods. Outlier thresholds (sum of the distances to all other arrays, Kolmogorov-Smirnov statistic Ka and Hoeffding's statistic Da) were calculated by the package based on the array signal intensity values.

We excluded probes not expressed in at least three arrays with detection *p*-values < 0.05. Pre-processing and probe filtering included background correction as described in (Shi et al., 2010b) using built-in negative controls and VSN normalization. A second procedure based on quantile normalization (limma neqc function) was used to test the main results of the differential expression analysis. We used the package illuminaHumanv4.db v1.26.0 as well as the manifest file for Illumina HumanHT-12v4 to annotate gene expression probes. We excluded probes from further analysis if probe sequences mapped to more than one genomic location; annealed at regions with SNPs present or mapped to non-autosomal locations (illuminaHumanv4.db v1.26.0). When mapping cytokines to their corresponding mRNA transcripts we found that only *IL-10* (ILMN_1674167) did not overlap known SNPs. Our final results filtered probes overlapping SNPs (Supplementary Table 1), but exclusion of these did not materially alter results. For comparison purposes, we present all cytokine transcripts (Fig. 3). Principal components analysis (PCA) was performed with R's prcomp function with scaling and centering.

We performed differential expression comparisons using limma v3.24.15 with linear models fit with empirical Bayes analysis (Smyth, 2004). The primary comparison was a difference in difference estimator (per gene expression probe). We tested for linear or quadratic effects of vitamin D on expression and on the absolute change in expression. Additionally, we performed a linear mixed model analysis with the R package lmerTest (Bates et al., 2015), using person-specific random effects to account for between-person expression heterogeneity and fixed effects for time and time interacted with 25(OH)D levels. To account for unknown confounders, we analyzed gene expression differences within time-points after correcting for the first 10 surrogate variables using the R packages SVA (Leek et al., 2012) and SmartSVA (Chen et al., 2017). We present the difference in difference comparisons but did not find significant changes using other statistical approaches for the main analysis.

#### 2.2.4 Expression Quantitative Trait Loci (eQTL) Analysis

We used an additive linear model as implemented in the R package MatrixEQTL v2.1.1 (Shabalin, 2012) with inclusion of principal components (PCs) from gene expression samples as covariates. We determined the number of PCs to correct for by running eQTL analyses with increasing numbers of PCs until the number of eQTL associations was maximized (Fairfax et al., 2014). Statistics and plots were carried out at the probe level. We used dbSNP human build 146, probe genomic locations as provided by Illumina, and *p*-value thresholds at <1e-8 for trans and <1e-5 for cis. We used MatrixEQTL's calculation of the false discovery rate (FDR) based on the Benjamini-Hochberg procedure. Vitamin D response eQTLs were defined using fold change values without a cut-off threshold as input (instead of gene expression values) for association with SNPs (FDR < 5%) after correcting for PCs per group. The maximum number of PCs to correct for was based on independent eQTL analyses of baseline and 12-months samples from gene expression values. We used R core packages, biglm (v. 0.9–1) and gvlma (v. 1.0.0.2) to regress PCs from gene expression values. We did not correct for population stratification as genomic inflation was low (1.01, based on median chi square) and unlikely to reflect population stratification (Yang et al., 2011, Bulik-Sullivan et al., 2015). Similar to the gene expression analysis, we tested for errors in the eQTL pipeline. Here we tested our results directly as comparable experiments have been done previously in whole blood samples (Westra et al., 2013). Despite differences in sample size, genotyping platforms and number of SNPs tested we chose a conservative genomic interval overlap test. We used the tool GAT (Heger et al., 2013) with the mappable genome as background and genomic intervals defined as plus and minus 1000 nucleotides for each SNP and ran the analysis of overlap between our results and (Westra et al., 2013) with 1000 permutations to obtain empirical *p*-values. Linkage disequilibrium (LD) clumping was performed using PLINK version 1.90 (Purcell et al., 2007) based on HapMap 3 (release 2) CEU population for eQTL SNPs under FDR 5% with an r^2^ threshold of 0.1, distance threshold of 10 kb and p-value of 0.0001.

#### 2.2.5 Statistical Analysis of Circulating Cytokines

We imputed missing data using multiple imputation methods with 50 datasets, a maximum iteration of 50 and predictive mean matching (R packages mice v2.30 and miceadds v2.4–12) (Van Buuren and Groothuis-Oudshoorn, 2011). No variable had >5% missing values. We performed analysis of covariance on each of the log natural transformed values of plasma levels of IFN-γ, IL-10, IL-8, IL-6 and TNF-α, accounting for the same confounders as in the genotype-25(OH)D analysis and including baseline values for every case. Linear regression summary tables presented were processed with the R package stargazer v2.3.1.

#### 2.2.6 General Software and Plotting

R packages were run with R 3.2.4 (R Core Team, 2016). Custom scripts, sqlite3 (v. 3.13.0) and data.table (v. 1.9.6) were used for data processing. Figures were generated using package specific functions (limma and MatrixEQTL) or with ggplot2 (v. 2.1.0) and R's base plotting. Supplementary Fig. 1 was plotted using code from (Anderson et al., 2010).

## 3 Results

### 3.1 Effects of Vitamin D Supplementation on Gene Expression

**Fig. 1:**
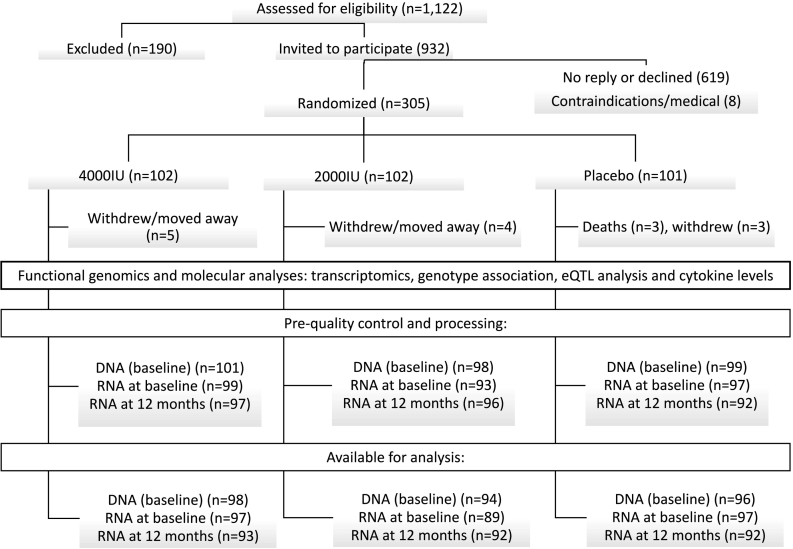
CONSORT diagram for the functional genomics analysis of participants in the BEST-D study. eQTL: expression quantitative trait loci.

Following sample processing and quality control, genome-wide gene expression data on 16,760 probes (12,910 genes) were available for 298 of 305 participants who were randomized to the trial (560 samples, of which 262 had both baseline and 12 month samples) (Fig. 1). The mean age of study participants was 72 years at randomization, 51% were male and 12% reported prior use of vitamin D supplements (≤400 IU of vitamin D_3_ daily) (Clarke et al., 2015). Compliance with instructions to take vitamin D supplements or placebo was high, with 90% (4000 IU), 92% (2000 IU) and 85% (placebo) reporting taking the capsules on all or most days at 12 months (Hin et al., 2017, Clarke et al., 2015). The overall mean plasma level of 25(OH)D was 50 nmol/L (standard error [SE] 1.04) at baseline and treatment was associated with mean plasma levels of 25(OH)D of 136 (3.94), 106 (2.55) and 50 (1.68) among those allocated to 4000 IU, 2000 IU, and placebo, after 12 months of treatment (unadjusted levels) (Table 1).

**Table 1:** Basic characteristics, baseline and 12 month values following vitamin D supplementation.

	Mean (SD)			sem			CI95% lower, upper			Change from baseline (mean, SD)			p-value		
	Placebo	2000 IU	4000 IU	Placebo	2000 IU	4000 IU	Placebo	2000 IU	4000 IU	Placebo	2000 IU	4000 IU	Placebo	2000 IU	4000 IU
Total n	101	102	102	–	–	–	–	–	–	–	–	–	–	–	–
Female n	49	51	50	–	–	–	–	–	–	–	–	–	–	–	–
Age	71.58 (5.64)	71.80 (6.15)	71.30 (5.52)	0.56	0.61	0.55	66.24, 76.92	66.46, 77.14	65.96, 76.64	–	–	–	–	–	–
BMI	28.01 (4.65)	27.37 (4.16)	27.22 (4.72)	0.46	0.41	0.47	22.67, 33.35	22.03, 32.71	21.88, 32.56	–	–	–	–	–	–
25(OH)D baseline	47.14 (14.62)	54.85 (22.61)	48.75 (15.16)	1.45	2.25	1.50	41.80, 52.48	49.51, 60.19	43.41, 54.09	–	–	–	–	–	–
25(OH)D 12 months	50.31 (16.34)	105.80 (25.25)	136.25 (38.81)	1.68	2.55	3.94	44.97, 55.65	100.46, 111.14	130.91, 141.59	3.50 (7.07)	50.94 (21.93)	87.07 (35.18)	5.50E-06	4.69E-41	6.65E-43
IFNg baseline	1.30 (0.70)	1.50 (0.94)	1.42 (0.78)	0.07	0.09	0.08	−4.04, 6.64	−3.84, 6.84	−3.92, 6.76	–	–	–	–	–	–
IFNg 12 months	1.30 (0.73)	1.53 (0.84)	1.42 (0.81)	0.07	0.08	0.08	−4.04, 6.64	−3.81, 6.87	−3.92, 6.76	0.00 (0.74)	0.07 (0.94)	0.02 (0.91)	0.97	0.48	0.79
IL10 baseline	−1.53 (1.12)	−1.40 (1.10)	−1.58 (0.98)	0.11	0.11	0.10	−6.87, 3.81	−6.74, 3.94	−6.92, 3.76	–	–	–	–	–	–
IL10 12 months	−1.40 (0.97)	−1.29 (0.96)	−1.43 (0.87)	0.10	0.10	0.09	−6.74, 3.94	−6.63, 4.05	−6.77, 3.91	0.12 (0.80)	0.16 (0.92)	0.19 (0.87)	0.14	0.10	0.04
IL6 baseline	−0.71 (0.67)	−0.60 (0.73)	−0.70 (0.79)	0.07	0.07	0.08	−6.05, 4.63	−5.94, 4.74	−6.04, 4.64	–	–	–	–	–	–
IL6 12 months	−0.72 (0.67)	−0.58 (0.75)	−0.61 (0.66)	0.07	0.08	0.07	−6.06, 4.62	−5.92, 4.76	−5.95, 4.73	0.01 (0.76)	0.03 (0.64)	0.12 (0.67)	0.94	0.59	0.07
IL8 baseline	1.22 (0.58)	1.28 (0.81)	1.31 (0.68)	0.06	0.08	0.07	−4.12, 6.56	−4.06, 6.62	−4.03, 6.65	–	–	–	–	–	–
IL8 12 months	1.14 (0.88)	1.34 (0.75)	1.34 (0.69)	0.09	0.08	0.07	−4.20, 6.48	−4.00, 6.68	−4.00, 6.68	−0.07 (0.88)	0.06 (0.83)	0.04 (0.57)	0.44	0.46	0.51
TNFa baseline	0.58 (0.33)	0.59 (0.40)	0.59 (0.36)	0.03	0.04	0.04	−4.76, 5.92	−4.75, 5.93	−4.75, 5.93	–	–	–	–	–	–
TNFa 12 months	0.57 (0.45)	0.59 (0.42)	0.60 (0.34)	0.05	0.04	0.03	−4.77, 5.91	−4.75, 5.93	−4.74, 5.94	−0.02 (0.37)	0.02 (0.34)	0.01 (0.27)	0.53	0.61	0.65
IFNg baseline (mRNA)	7.04 (0.20)	7.03 (0.21)	7.03 (0.18)	0.02	0.02	0.02	1.70, 12.38	1.69, 12.37	1.69, 12.37	–	–	–	–	–	–
IFNg 12 months (mRNA)	7.00 (0.15)	7.03 (0.23)	7.02 (0.22)	0.02	0.02	0.02	1.66, 12.34	1.69, 12.37	1.68, 12.36	−0.05 (0.18)	−0.01 (0.27)	−0.02 (0.22)	0.02	0.63	0.42
IL10 baseline (mRNA)	6.87 (0.08)	6.86 (0.09)	6.88 (0.08)	0.01	0.01	0.01	1.53, 12.21	1.52, 12.2	1.54, 12.22	–	–	–	–	–	–
IL10 12 months (mRNA)	6.86 (0.08)	6.87 (0.07)	6.87 (0.08)	0.01	0.01	0.01	1.52, 12.2	1.53, 12.21	1.53, 12.21	−0.01 (0.11)	0.01 (0.11)	−0.01 (0.12)	0.39	0.49	0.36
IL6 baseline (mRNA)	6.93 (0.09)	6.93 (0.09)	6.93 (0.08)	0.01	0.01	0.01	1.59, 12.27	1.59, 12.27	1.59, 12.27	–	–	–	–	–	–
IL6 12 months (mRNA)	6.93 (0.07)	6.93 (0.09)	6.93 (0.08)	0.01	0.01	0.01	1.59, 12.27	1.59, 12.27	1.59, 12.27	0.00 (0.11)	0.00 (0.12)	0.00 (0.12)	0.82	0.82	0.94
IL8 baseline (mRNA)	8.06 (0.61)	8.10 (0.61)	7.99 (0.66)	0.06	0.06	0.07	2.72, 13.40	2.76, 13.44	2.65, 13.33	–	–	–	–	–	–
IL8 12 months (mRNA)	7.91 (0.61)	7.93 (0.65)	7.90 (0.67)	0.06	0.07	0.07	2.57, 13.25	2.59, 13.27	2.56, 13.24	−0.13 (0.7)	−0.17 (0.73)	−0.08 (0.79)	0.09	0.04	0.34
TNFa baseline (mRNA)	8.30 (0.28)	8.31 (0.24)	8.32 (0.24)	0.03	0.03	0.02	2.96, 13.64	2.97, 13.65	2.98, 13.66	–	–	–	–	–	–
TNFa 12 months (mRNA)	8.35 (0.24)	8.34 (0.24)	8.36 (0.24)	0.02	0.03	0.02	3.01, 13.69	3.00, 13.68	3.02, 13.70	0.05 (0.28)	0.03 (0.28)	0.04 (0.27)	0.12	0.27	0.17

Arithmetic mean, standard deviation (SD), standard error of the mean (sem), 95% confidence intervals (CI95%) and two-sided, univariate, paired t-test p-values shown (baseline versus 12 months within each arm).

Values are for observed data only. Values presented are age in years, body mass index (BMI) in kg/m^2, 25(OH)D in nmol/L.

Circulating cytokine values are natural logarithm transformed. mRNA values are VSN normalized.

P-values are not adjusted for confounding, baseline values or multiple testing.

Regression models and further results are shown in Supplementary Table 1 (gene expression), Supplementary Table 2 (circulating cytokines), Supplementary Table 3 (genetic association) and Supplementary Table 4 (expression QTL).

Regression models and further results are shown in Supplementary Table 1 (gene expression), Supplementary Table 2 (circulating cytokines), Supplementary Table 3 (genetic association) and Supplementary Table 4 (expression QTL).

**Fig. 2:**
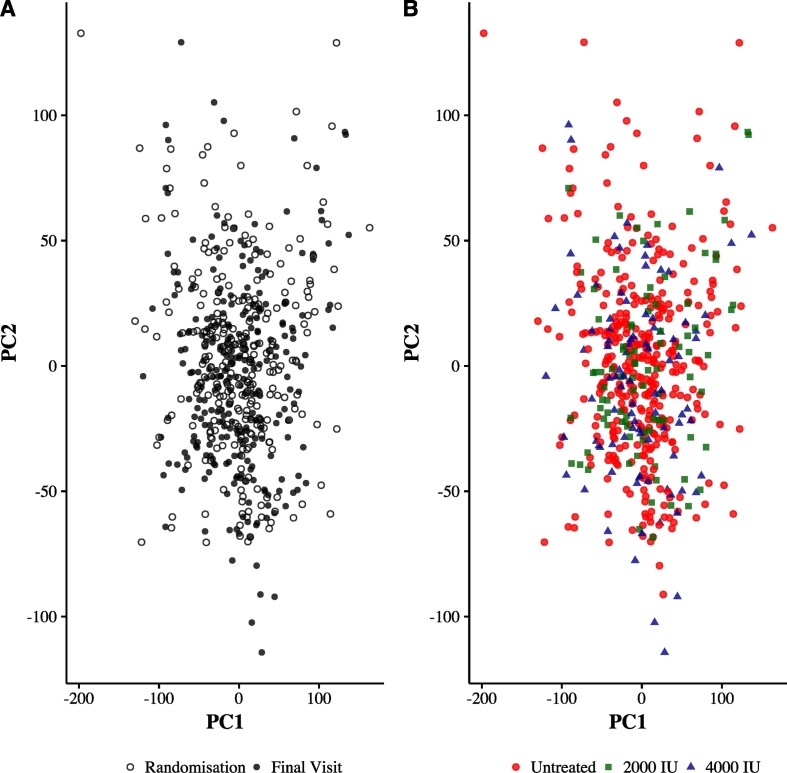
Global gene expression by allocated treatment with either vitamin D or placebo supplementation was assessed using principal components analysis of variance. Principal components were calculated based on post-QC and VSN normalized gene expression signals. PC1 and PC2 (representing 15% and 8.5% of variance, respectively) are shown and colored according to time-point (final visit at 12 months or at randomization [baseline], panel A) and separately by treatment group (placebo, 2000 IU or 4000 IU, panel B).

We first performed PCA to visualize the relationship between samples based on gene expression values. Considering all samples or paired samples within treatment or placebo groups, we found no visual evidence of clustering using up to the first 13 PCs (accounting for 41% of variance) (Fig. 2, Supplementary Fig. 2A and Supplementary Figs. 3–6). The top 100 PCs accounted for 60% of the total variance (Supplementary Fig. 3).

**Fig. 3:**
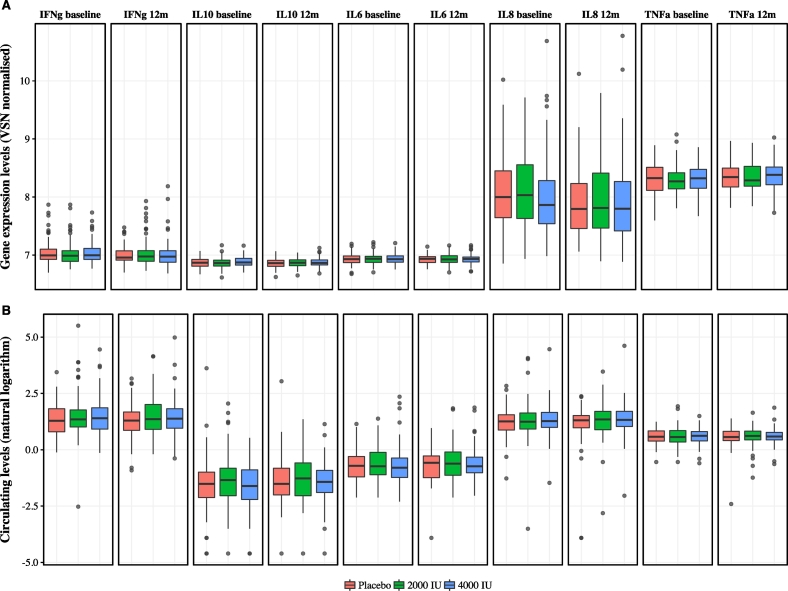
Boxplots of plasma cytokine levels and their corresponding transcripts before and after 12 months of vitamin D supplementation by allocated treatment. Gene expression (RNA) and circulating cytokine (protein) levels did not show changes after supplementation when accounting for baseline levels, known confounders and placebo (see Methods). VSN normalized mRNA levels (top row) and log-transformed protein levels (bottom row) of cytokines in whole-blood at baseline and 12 months (y-axis) for each trial arm (x-axis: red (left) = placebo, green (middle) = 2000 IU, blue (right) = 4000 IU).

**Fig. 4:**
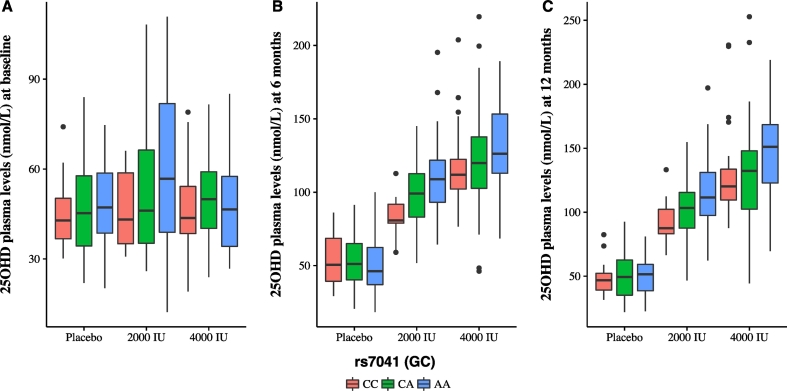
Genetic association analysis following vitamin D supplementation. Boxplots of rs7041 (*GC)* showing post-supplementation plasma levels of 25(OH)D by allocated treatment. Associations with rs7041 are statistically significant at both 6 (*p* = 0.001, panel B) and 12 months (*p* = 0.023, panel C) at 2000 IU using permutation to derive empirical *p*-values after adjusting for baseline 25(OH)D levels and other relevant variables (see Methods and Supplementary Table 3). x-axis: genotypes, red (left) = CC, green (middle) = CA and blue (right) = AA. y-axis: 25(OH)D circulating levels (nmol/L) at baseline (A), 6 months (B) and 12 months (C). (For interpretation of the references to colour in this figure legend, the reader is referred to the web version of this article.) Genetic association analysis following vitamin D supplementation. Boxplots of rs7041 (*GC)* showing post-supplementation plasma levels of 25(OH)D by allocated treatment. Associations with rs7041 are statistically significant at both 6 (*p* = 0.001, panel B) and 12 months (*p* = 0.023, panel C) at 2000 IU using permutation to derive empirical *p*-values after adjusting for baseline 25(OH)D levels and other relevant variables (see Methods and Supplementary Table 3). x-axis: genotypes, red (left) = CC, green (middle) = CA and blue (right) = AA. y-axis: 25(OH)D circulating levels (nmol/L) at baseline (A), 6 months (B) and 12 months (C).

We next formally tested the effects of vitamin D supplementation on genome-wide gene expression considering significantly differentially expressed probes at an FDR < 5% for any fold change in an unadjusted model. As expected after successful randomization, we did not observe any significant differences in gene expression when comparing allocation groups at baseline (placebo baseline, 2000 IU baseline and 4000 IU baseline) (Supplementary Table 1).

The pre-defined primary outcome sought to determine differences in gene expression in response to any dose of vitamin D compared with placebo. We compared differences in transcriptome among participants allocated to vitamin D (4000 IU or 2000 IU) at 0 vs 12 months versus those allocated to placebo. Difference in difference analysis was estimated using (expression gene A following vitD_12months_ − expression gene A vitD_baseline_) − (expression gene A placebo_12months_ − expression gene A placebo_baseline_). We found that 375 probes (for 4000 IU minus placebo using the difference in difference estimate as above) and 329 probes (for 2000 IU minus placebo) were significantly differentially expressed following vitamin D supplementation (unadjusted *p*-value < 0.05), but none remained significant after taking account of multiple comparisons (FDR < 5%) (Supplementary Table 1). Use of quantile or variance stabilizing normalization (VSN) methods for data processing did not materially alter the results (Supplementary Fig. 7, see Methods for details). The placebo group, with both baseline and 12 month sampling, is a stringent control that was used to account for the effect of time, placebo itself, technical artefacts or other sources of variation. After controlling for relevant confounders, there were no differences in gene expression between individuals allocated vitamin D versus those allocated placebo. It is possible that reliable detection of gene expression in response to vitamin D supplementation may require a larger sample size (Supplementary Fig. 7).

Subsequent analyses considered less conservative comparisons as pre-specified (see data analysis protocol, Supplementary Appendix). We conducted two group comparisons of each arm (4000 IU at 12 months vs 4000 IU at baseline; and separately, 2000 IU at 12 months vs 2000 IU at baseline), but did not find significant differences in gene expression (FDR < 5%). We also compared 12-month samples (4000 IU at 12 months vs placebo at 12 months; 2000 IU at 12 months vs placebo at 12 months), but did not observe any significant differences (FDR < 5%). Likewise, paired analysis for each of these comparisons did not yield significant differences (Supplementary Table 1).

To maximize power, we combined all vitamin D allocated individuals before and after treatment (2000 IU plus 4000 IU vs their baseline samples, unpaired), but did not detect significant differences in expressed probes (FDR < 5%). Paired sample comparisons (*n* = 186, joint 2000 IU and 4000 IU) for this grouping indicated some significant differences following vitamin D supplementation (143 probes at <5% FDR; 292 probes at FDR < 10%, fold change range 0.83–1.12, Supplementary Table 1). However, neither of these analyses took account of the differences in the placebo group and, hence, were less robust than the analyses of differences and random effects detailed above.

We hypothesized that differences in transcriptomes would be more evident when comparing individuals with low plasma levels of 25(OH)D. We compared those with pre-treatment plasma levels of 25(OH)D < 50 nmol/L versus those with 25(OH)D > 50 nmol/L (124 vs 159, respectively, unadjusted model) regardless of the allocated treatment, and separately, cases of more extreme change (<25 nmol/L vs >75 nmol/L 25(OH)D, 13 vs 30, respectively). Likewise, allocation to vitamin D had no significant differences in gene expression (FDR < 5%, Supplementary Table 1). We repeated such comparisons using subsets with paired samples before and after treatment among individuals who at baseline were deficient (<50 nmol/L and separately for those with <25 nmol/L), but did not detect any significant differences in either subgroup (FDR < 5%, Supplementary Table 1). Finally, although we did not pre-specify it, we selected individuals whose change (delta) in 25(OH)D levels was high (vitD_12months_ − vitD_baseline_), regardless of allocated treatment. Although this analysis is more likely to be confounded it provided more power as more individuals with greater differences in plasma vitamin D in response to supplementation were included. We chose the median (+44.79 nmol/L) as this yielded the highest change in the maximum number of individuals (*n* = 145). As expected, none of the placebo group had a difference of this magnitude (median: +2.58 nmol/L). In paired analysis we found five genes significantly different at an FDR of <5% (Supplementary Table 1), but the effect sizes for these genes were small (fold change range: 0.81–1.12) and further work is needed to replicate such associations in other trial populations. Finally, given that gender specific effects have been noted previously (Pasing et al., 2017), we analyzed segregated samples (women only and men only) but did not find significant differences in paired analysis (women *n* = 144, men *n* = 141).

### 3.2 Changes in Plasma Cytokine Levels Following Vitamin D Supplementation

In addition, we assessed whether supplementation with vitamin D had any significant effect on plasma levels of cytokines (Table 1). Consistent with lack of effect on gene expression results in the present and other previous studies (Ter Horst et al., 2016), we did not identify any significant effects of vitamin D supplementation on plasma levels on cytokines. Likewise, we found no significant correlations between plasma levels of 25(OH)D and plasma levels of IFN-γ, IL-10, IL-8, IL-6 or TNF-α at 12 months (Supplementary Figs. 8 and 9). Multivariate regression models testing the effect of supplementation after 12 months for either dose of vitamin D on changes in plasma cytokine levels (IFN-γ, IL-10, IL-8, IL-6 or TNF-α) after accounting for known confounders and baseline values did not show significant changes (Fig. 3 and Supplementary Table 2).

### 3.3 Individual Responsiveness to Vitamin D Supplementation: Impact of Genotype

This study lacked power to assess differences in the effects of treatment with vitamin D by differences in genome-wide genetic variation. We restricted our analysis to SNPs with prior evidence of association with plasma 25(OH)D levels from population GWAS (Wang et al., 2010) (rs12794714 [*CYP2R1*], rs2282679 [*GC*], rs7041 [*GC*] and rs7944926 [*DHCR7/NADSYN1*]) for which we had genotyping data available. Previous studies in twins suggested that summer levels of 25(OH)D were not strongly influenced by genetic variation (Orton et al., 2008, Karohl et al., 2010). Our pre-specified analysis assessed the hypothesis that genotype may modulate the response to vitamin D supplementation. We analyzed 25(OH)D levels following treatment with measurements at 6 and 12 months, using baseline vitamin D and other variables as covariates in a linear regression model (see Methods). We found that rs7041 (located on chromosome 4, *GC*) was significantly associated with response to vitamin D treatment with low dose vitamin D (2000 IU) at 6 and 12 months (permuted *p* values 0.001 and 0.023 respectively) (Fig. 4 and Supplementary Table 3). At high dose (4000 IU), we found no significant effects of rs7041 (or other SNPs). Although higher doses may abrogate the genetic effect, larger studies with greater statistical power are needed to confirm or refute this hypothesis.

We next considered whether there was evidence that genetic determinants of gene expression were modulated by supplementation with vitamin D by adopting a genetical genomics approach. Genetic variation is known to be an important determinant of individual gene expression and to be highly context-specific (Gibson et al., 2015). We hypothesized that genetic variation may be an important contributor to individual gene expression differences in response to vitamin D. Although our study did not detect differences in gene expression after supplementation, we carried out an expression quantitative trait analysis as pre-specified given that individual level effects on gene expression dependent on genotype may still occur. Following sample processing and quality control, we analyzed genotyping data on 497,136 variants for 288 individuals. We tested for evidence of association with gene expression using an additive linear model for 14,972 probes, including the top PCs as covariates after maximizing for cis-eQTLs in each group (see Methods). We defined expression associated SNPs (eSNPs) as *cis*-eSNPs (those located within 1 Mb of the gene expression probe) or *trans*-eSNPs (located >1 Mb of the gene expression probe). At baseline, we found 31,568 cis-eQTLs (18,245 LD clumped index SNPs and 3278 unique probes) and 34,254 (19,345 LD clumped index SNPs and 3390 unique probes) at 12-months (unique SNP-probe pairs, 2000 IU and 4000 IU groups jointly to maximize sample size, FDR < 5%) (Supplementary Table 4). There was a significant and positive overlap with comparable previously published data for whole blood eQTL (Westra et al., 2013) indicating high reproducibility despite large differences in sample size (1.75 fold-change overlap, q-value < 0.001). To investigate response eQTLs present in samples from vitamin D supplemented individuals, we performed an analysis that took into account both effect size (gene expression differences) and statistical significance by obtaining eQTLs from the fold changes between treated (joint 2000 IU and 4000 IU 12 months' supplementation) and their baseline values after correcting for PCs as outlined above. We found no significant associations involving response eQTLs (FDR < 10%, Supplementary Table 4, Supplementary Fig. 10).

## 4 Discussion

Overall, the present study demonstrated that allocation to high-dose oral vitamin D_3_ in 305 older people had no significant effect on gene expression or plasma levels of cytokines when measured after 12 months despite achieving significantly higher plasma 25(OH)D levels (Hin et al., 2017). To our knowledge, this is the largest randomized, placebo-controlled trial that assessed molecular changes and genetic effects following vitamin D supplementation.

Previous studies lacking appropriate randomization or use of placebo controls may have been confounded by time. Both seasonal and age related effects on gene expression have been reported previously (Dopico et al., 2015, de Magalhaes et al., 2009). Careful design and analysis are required; data analysis protocols can be pre-specified with blinding maintained. We demonstrated no significant effect of supplementation with either 2000 IU or 4000 IU of vitamin D on genome-wide gene expression or on plasma levels of IFN-γ, IL-10, IL-8, IL-6 and TNF-α after one year of supplementation. The results of the present study suggest that plasma levels of vitamin D after supplementation can be modified by genetic variation, in agreement with a recent trial in a different population (Yao et al., 2017).

The findings of the present study agree with the main results of a recent trial assessing the effect of vitamin D supplementation on gene expression in subjects with reduced glucose tolerance (Pasing et al., 2017). Interestingly, this study observed differences in sub-group analyses by gender and circulating vitamin D quintiles. The trial had a smaller sample size, focused on a different population and lacked baseline controls however.

The present study has several limitations. We did not collect samples within the first few hours or days following intervention and cannot exclude early transcriptomic changes. Indeed, other studies have observed changes in chromatin accessibility at candidate regions following vitamin D supplementation in peripheral blood mononuclear cells (Seuter et al., 2016). Similarly, other tissues and specific cell types may show differences not observable in whole blood samples. We cannot address whether inflammatory processes deplete plasma levels of vitamin D, but our results do not support the hypothesis that long-term supplementation modulates plasma levels of cytokines. The study population included Caucasian, community-dwelling older individuals. Younger individuals, those with particular diseases or different ethnicities may respond differently. Although we did not find differences for those with baseline vitamin D deficiency, our study was not designed to target this group. Larger studies may find significant differences, albeit of likely smaller effect, and focused studies may identify changes in specific cell types or observe differences in individuals with vitamin D deficiency.

This study demonstrated that genome-wide genetic variation, transcriptome and genetics of drug induced gene expression profiling can be integrated with biochemical and physiological measurements in the context of a randomized trial and may add important molecular and mechanistic insights. Interdisciplinary and collaborative efforts can guide the design and conduct of trials which require careful sample, data collection and analysis.

Further studies are needed to replicate the null associations and to assess why there were no long-term detectable differences after vitamin D treatment on gene expression. Physiological mechanisms that regulate the metabolism of vitamin D may achieve a steady state. Higher plasma levels of 25(OH)D may allow resources to be mobilized when needed. Changes in chromatin may better reflect effects of vitamin D supplementation (Carlberg et al., 2018). Upregulation of catabolic enzymes, unobserved confounding, and tissue specificity, among others, may account for the null findings. The present study highlights the difficulty in translating results from model organisms, in-vitro and ex-vivo studies and argues for further integration of molecular and clinical studies from in vivo observations.

The following are the supplementary data related to this article.

Supplementary Table 1: Differential gene expression comparisons in vitamin D trial. (A) Legend and description of all comparisons. (B) Main comparisons. (C) All comparisons (including those considered main [ST1B]). Supplementary Table 1

Supplementary Table 2: Summary of analysis of covariance of circulating cytokines. Supplementary Table 2

Supplementary Table 3: Genetic association results of candidate markers. Supplementary Table 3

Supplementary Table 4: Expression quantitative trait locus analysis. Supplementary Table 4

Supplementary Table 5: Vitamin D and Mendelian Randomization literature review Supplementary Table 5

Supplementary Table 6: Literature review of vitamin D clinical trials in the 12 months to June 12, 2017. Supplementary Table 6

Supplementary material 1 Image 1

Supplementary material 2. Image 2

Supplementary material 3. Image 3

## Role of the Funding Source

The study received funding from the British Heart Foundation, UK Medical Research Council and CTSU, University of Oxford. The British Heart Foundation (PG/12/32/29544) and British Heart Foundation Centre for Research Excellence provided partial funding for the study. Active and placebo vitamin D capsules were kindly donated by Tischcon Corporation (Westbury, New York, USA). The funders had no role in data collection, analysis, interpretation or writing of the report. All authors had access to all the data in the study. Trial registration: SRCTN Number 07034656; EudraCT Number 2011-005763-24.

## Author Contributions

AJBT and JCK conceived the study. AJBT, JA, RC and JCK designed the study. JA and RC are the principal investigators of the BEST-D trial. AJBT performed the analysis with input from AD, DS, AH and JE. KP, EL and MH performed experiments. JA, RC and JCK provided senior supervision. AJBT and JCK wrote the manuscript with contributions from all authors.

## Competing Interests

None.

## Data and Materials Availability

Gene expression data are available through ArrayExpress (E-MTAB-6246). Cytokine, phenotype and genotyping data are available from CTSU, University of Oxford through a material transfer agreement prior consent. All computational code used for processing and analysis is available at https://github.com/AntonioJBT/BEST_D.

## Funding

Medical Research Council, British Heart Foundation, Wellcome Trust, European Research Council and Clinical Trial Service Unit, Nuffield Department of Population Health, University of Oxford, Oxford, United Kingdom.

## Copyright

Open access article under the terms of CC BY.
