# Supplementary material for: Genomic Response to Vitamin D Supplementation in the Setting of a Randomized, Placebo-Controlled Trial
Source: eBioMedicine. 2018 Apr 10;31:133–42. doi: 10.1016/j.ebiom.2018.04.010 (PMC6013786; doi:10.1016/j.ebiom.2018.04.010)
Supplement: Supplementary file 7 — Supplementary material 1 [file mmc7.pdf]

Supplementary figures

Effect of vitamin D supplementation on biomarkers of inflammation and immune function: functional genomics analysis of the BEST-D trial

Antonio J. Berlanga-Taylor, Katherine Plant, Evelyn Lau, Andrew Dahl, Michael Hill, David Sims, Andreas Heger, Jonathan Emberson, Jane Armitage, Robert Clarke and Julian C. Knight

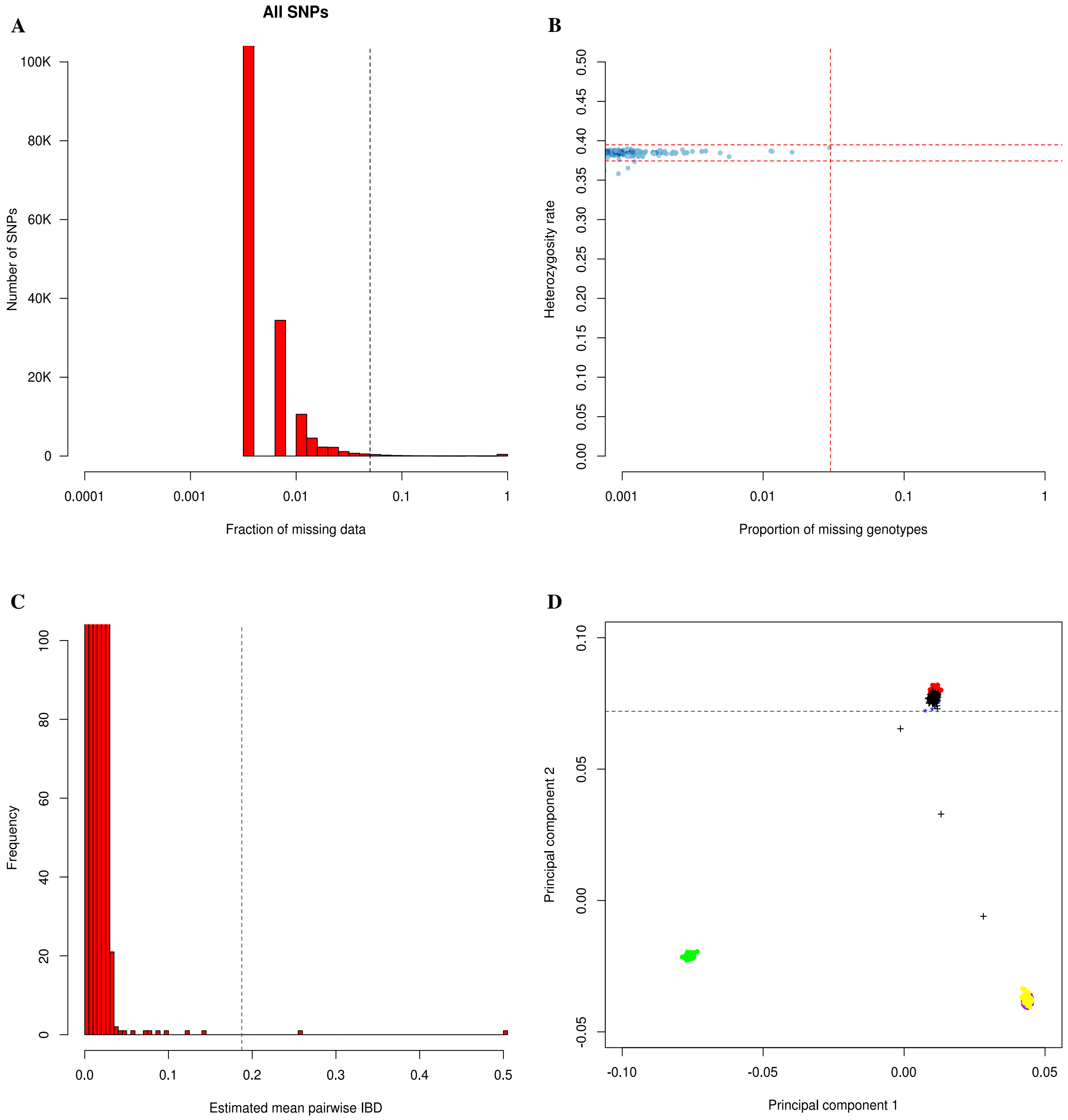

Supplementary Figure 1: Genotype data QC. (A) Distribution of missing genetic markers by sample. Markers were removed if the failure rate was >3% (dashed line). **(B)** Proportion of missing markers (x-axis) and heterozygosity rates (y-axis): Each dot is an individual (with colour shading for density). Samples were excluded if they fell outside the dashed lines (>3% genotype failure rate, +/- 3 SD heterozygosity rate). **(C)** Distribution of pairwise identity by descent (IBD). Dashed line corresponds to IBD of 0.185 (IBD = 1, monozygotic twins, IBD = 0.125, third-degree relatives). One individual of each pair of samples with higher values was removed. **(D)** Clustering by population ancestry using genome-wide data: BEST-D samples (black crosses) and HapMap3 reference samples (CEU red; CHB + JPT purple and yellow; YRI green). Samples with a PC2 score <0.072 (dashed line) were excluded.

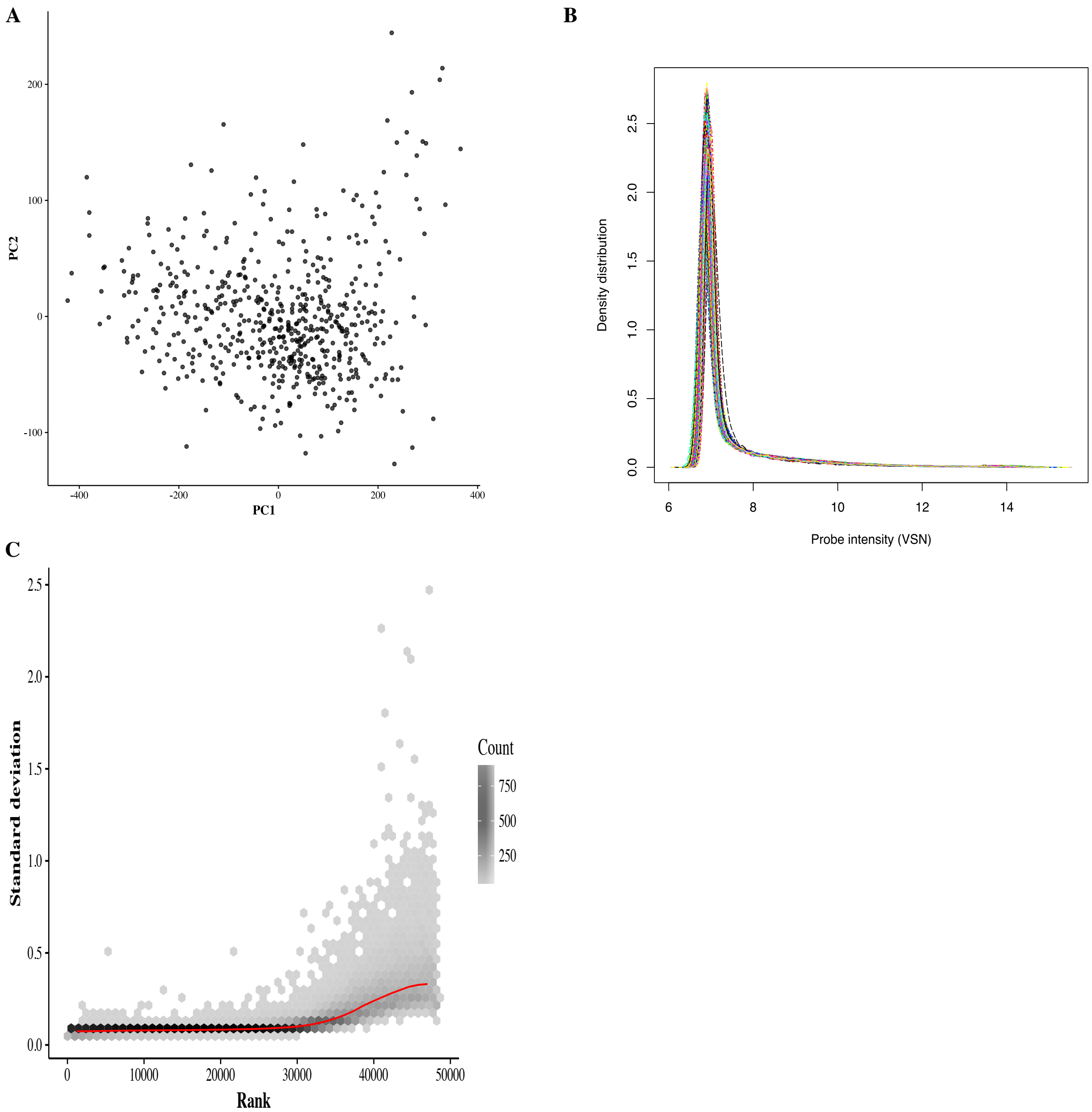

Supplementary Figure 2: Gene expression QC. (A) Visualisation of samples before processing: Principal component analysis of gene expression values before quality control and data processing (PC1 and PC2 represent 46% and 6% of variance respectively). **(B)** Density distribution of probe intensities of all samples after quality control and VSN processing. Each line represents one individual. **(C)** Standard deviation (SD) versus rank of the mean intensity per probe: Density plot of the SD of the intensities (y-axis) versus the rank of their mean (x-axis). Red dots represent the running median of the SD.

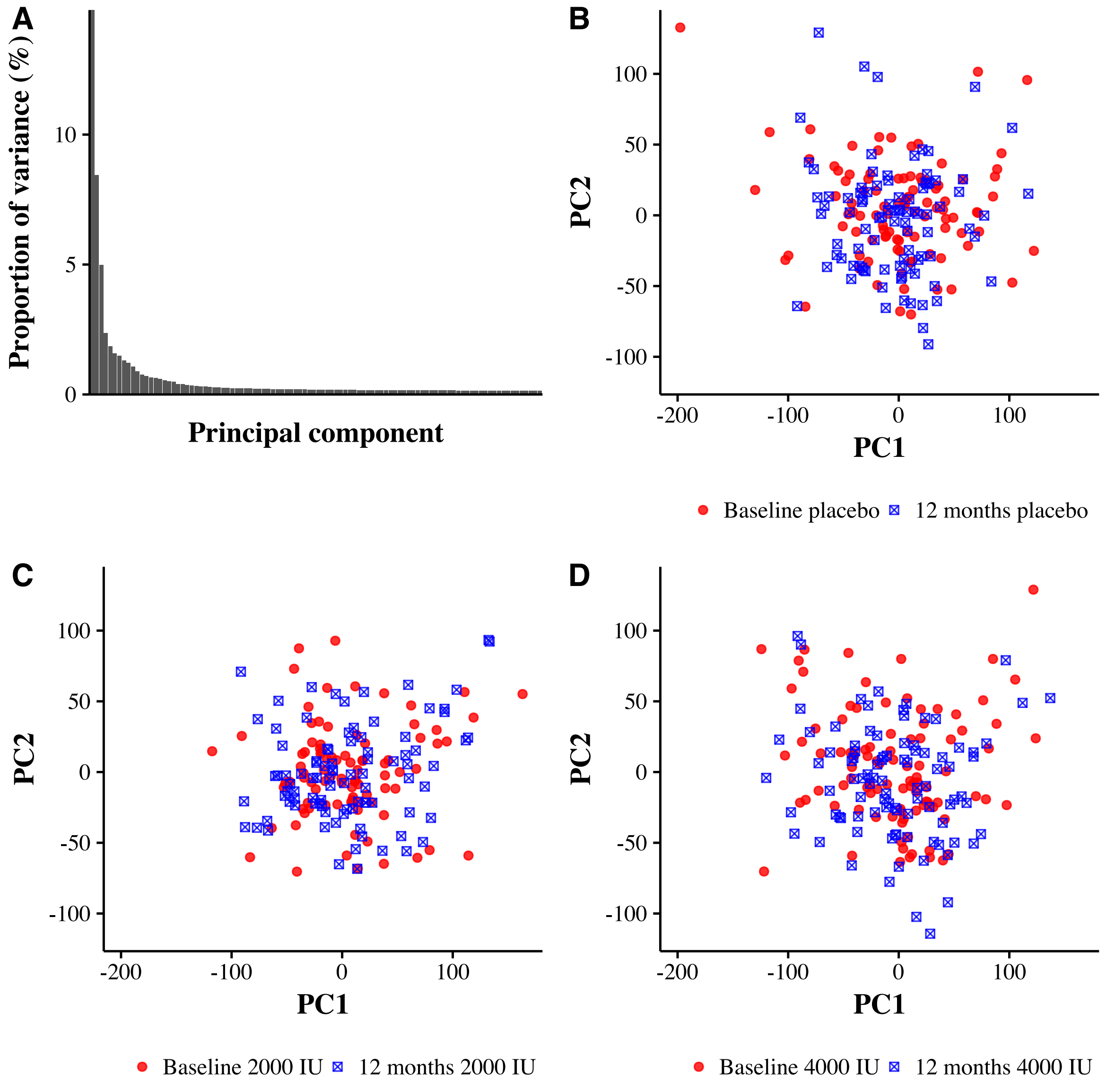

Supplementary Figure 3: Principal component analysis of gene expression values. Principal components (PC) were calculated based on post-QC and VSN normalised gene expression signals. Proportion of variance explained for the top 100 PCs (top left). Other panels: Each panel shows PC1 and PC2 according to time-point (final visit at 12 months or at randomisation [baseline]) and allocation arm (placebo, 2000 IU and 4000 IU).

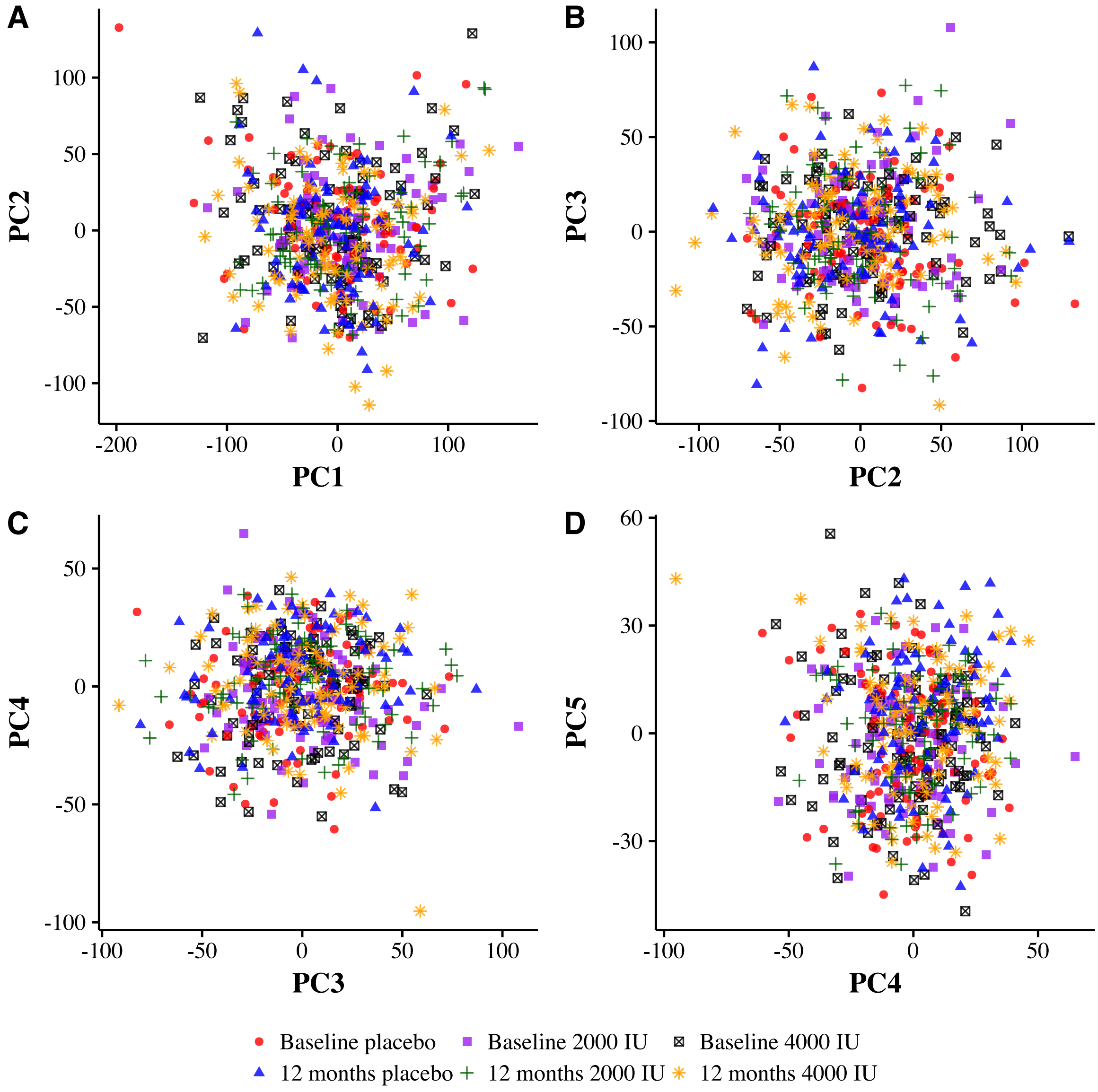

Supplementary Figures 4: Principal component analysis of gene expression values. Principal components (PC) were calculated based on post-QC and VSN normalised gene expression signals. PCs 1 to 13 shown according to time-point and allocation arm. Each panel shows all groups (baseline and 12 months for placebo, 2000 IU and 4000 IU).

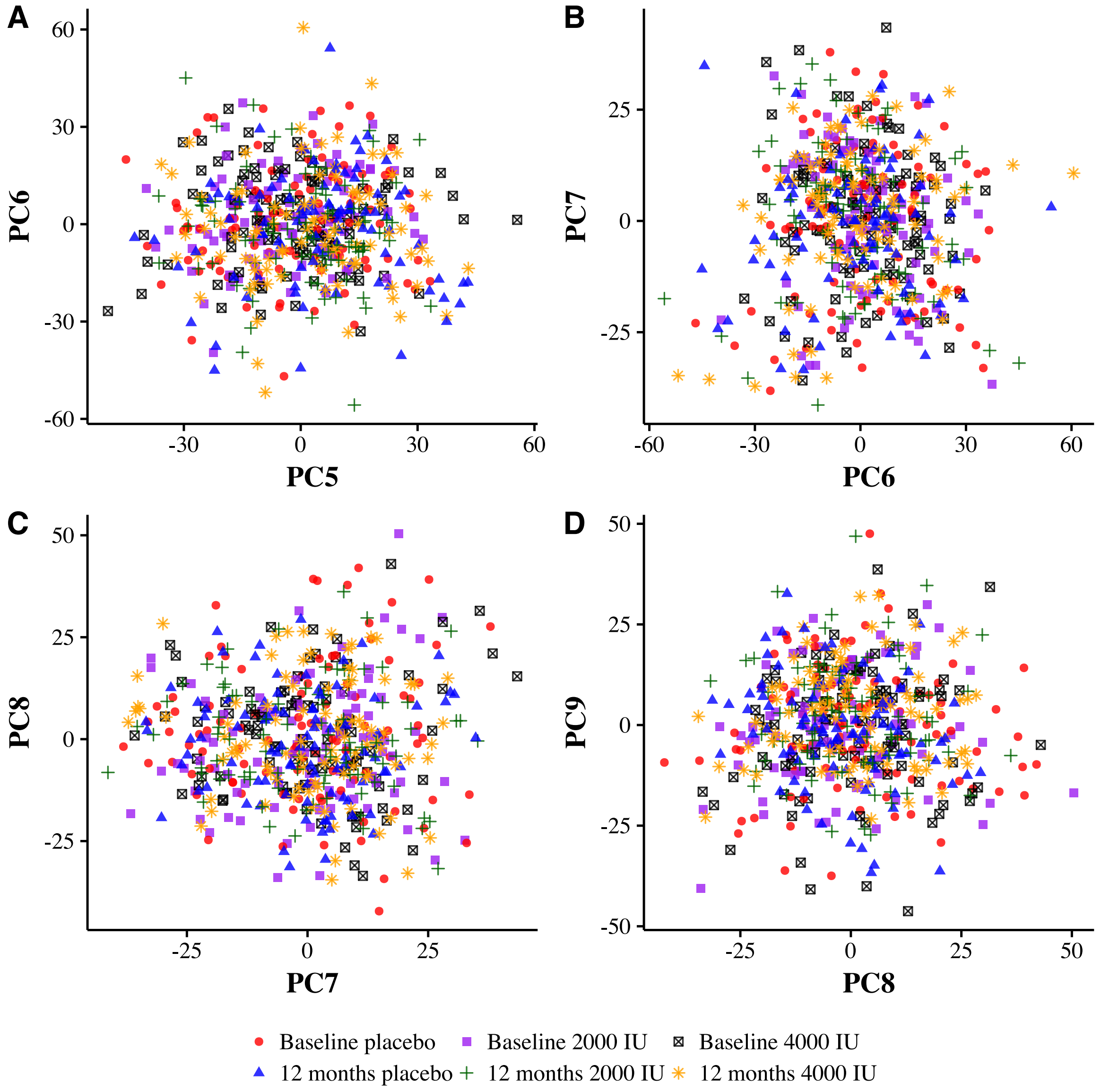

Supplementary Figures 5: Principal component analysis of gene expression values. Principal components (PC) were calculated based on post-QC and VSN normalised gene expression signals. PCs 1 to 13 shown according to time-point and allocation arm. Each panel shows all groups (baseline and 12 months for placebo, 2000 IU and 4000 IU).

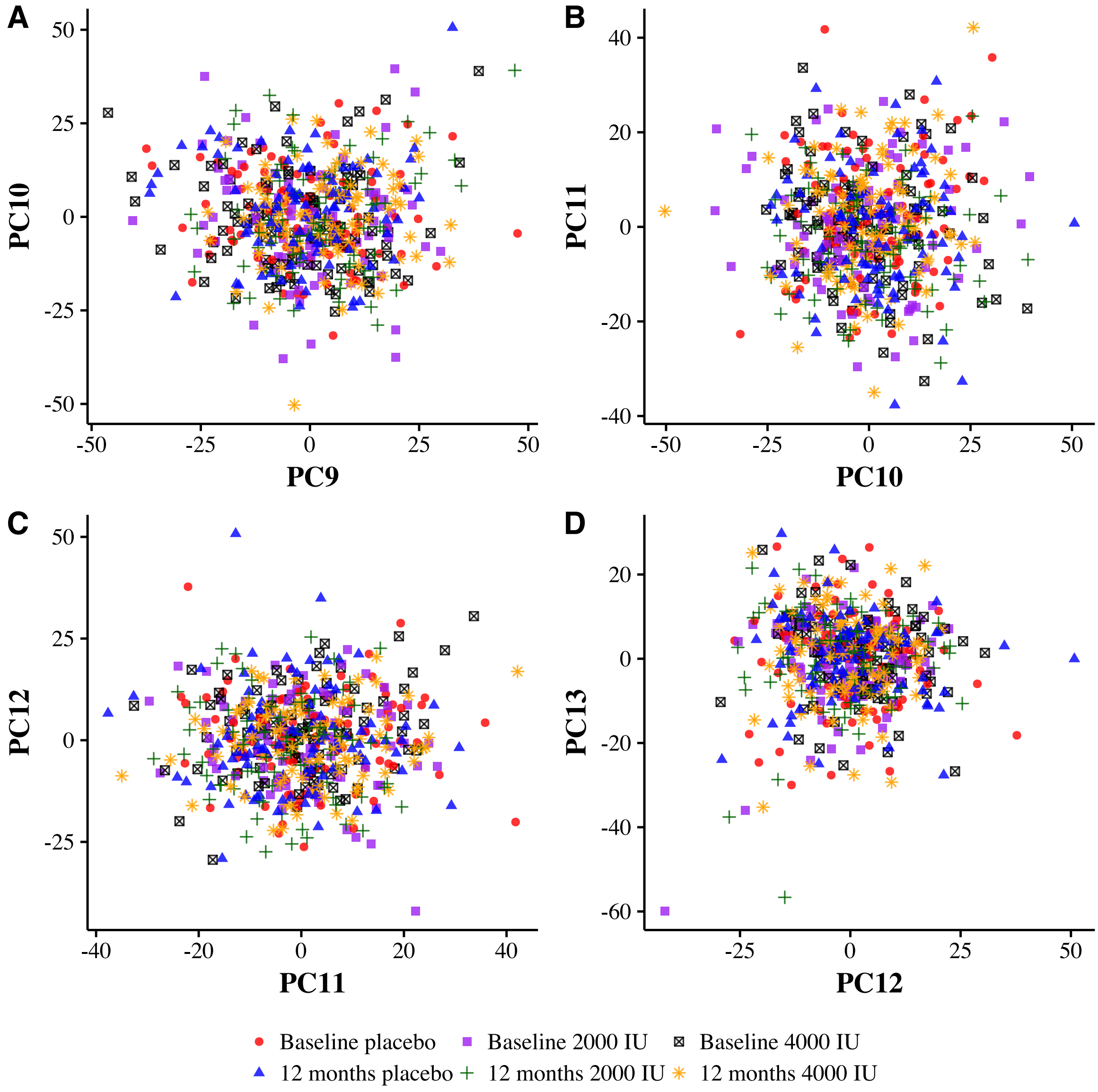

Supplementary Figures 6: Principal component analysis of gene expression values. Principal components (PC) were calculated based on post-QC and VSN normalised gene expression signals. PCs 1 to 13 shown according to time-point and allocation arm. Each panel shows all groups (baseline and 12 months for placebo, 2000 IU and 4000 IU).

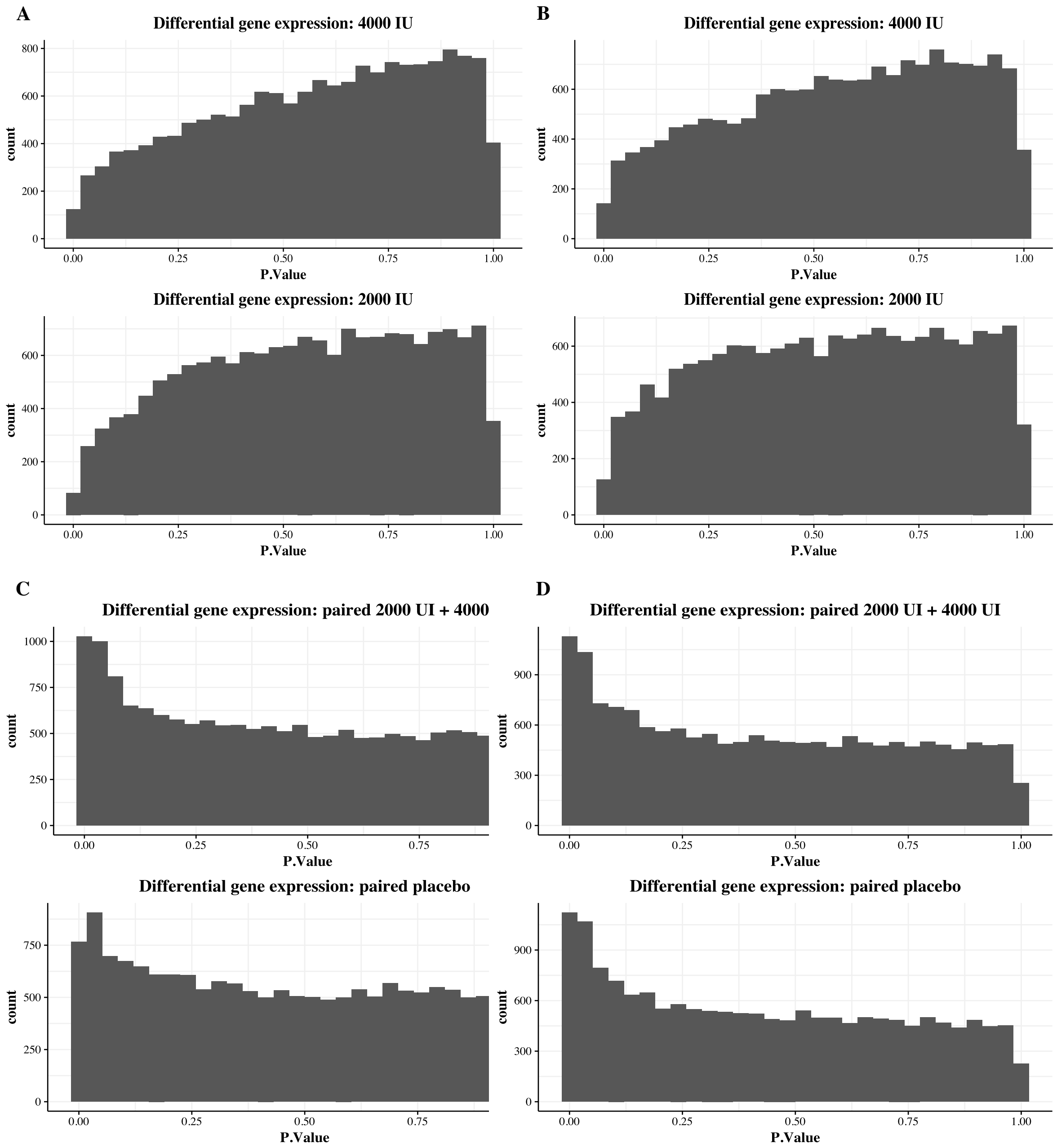

Supplementary Figure 7: Gene expression p-value distributions. Distribution of raw p-values of differential gene expression analysis (difference in difference estimates panels A and B; joint 2000 IU plus 4000 IU groups paired analysis [12 months vs baseline]; and paired placebo [12 months vs baseline] panels C and D) after quality control and processing with VSN (A and C) and quantile normalization (B and D). Increased power appears to change the shape of the distribution of p-values (plots A and C; B and D) and may point to increased variance after supplementation. Effects are too small to confidently detect and person-specific random effects analysis did not detect differences in gene expression after supplementation.

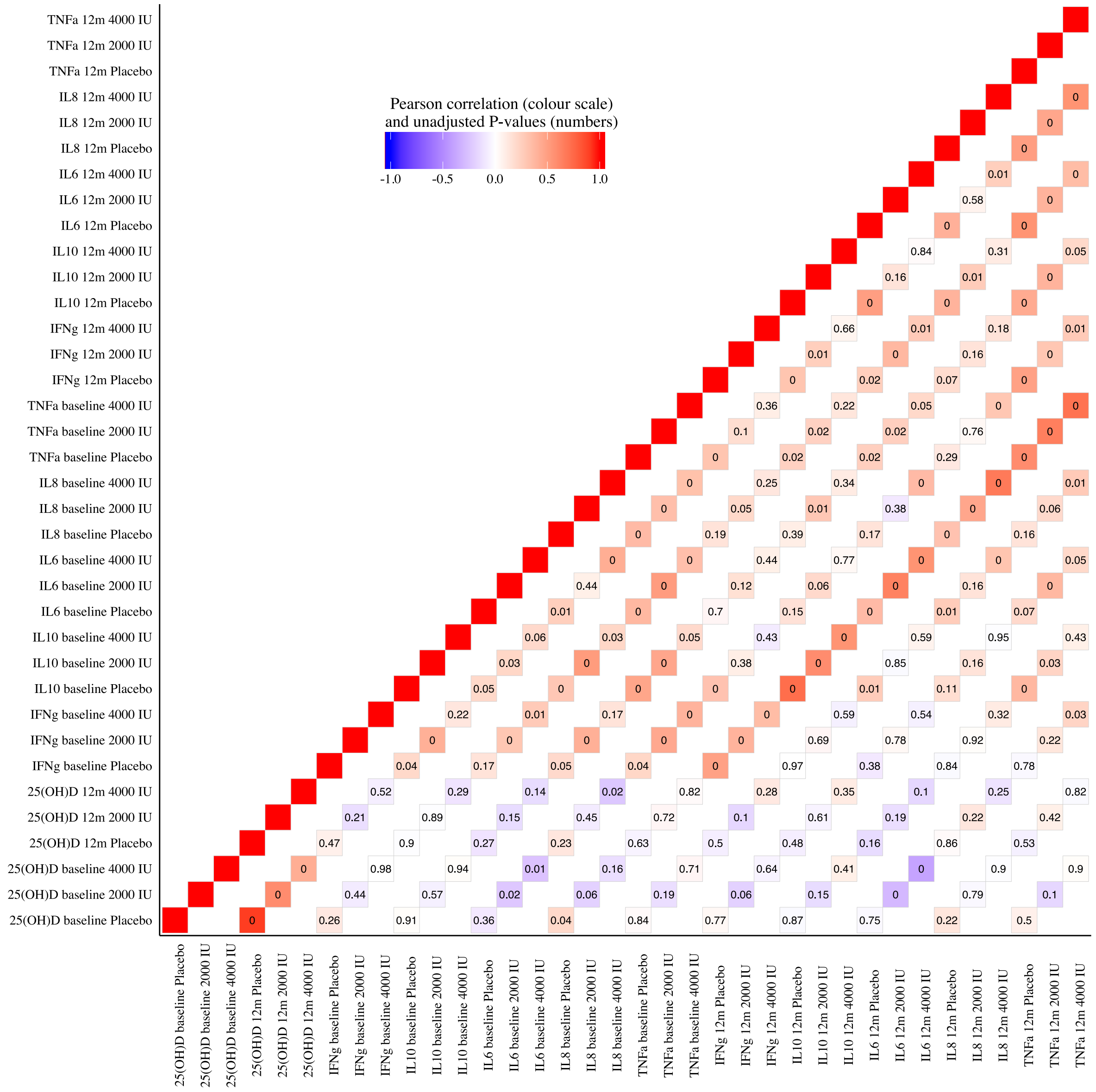

Supplementary Figure 8: Heatmap of circulating cytokines and vitamin D. Pairwise Pearson correlations of 25(OH)D and cytokine (protein) levels at baseline and 12 months by arm. Darker colours (blue negative, red positive) indicate higher correlation while white indicates zero. Unadjusted p-values are plotted within. P-values are rounded to 2 digits and ‘0’ values correspond to <2.2e-16.

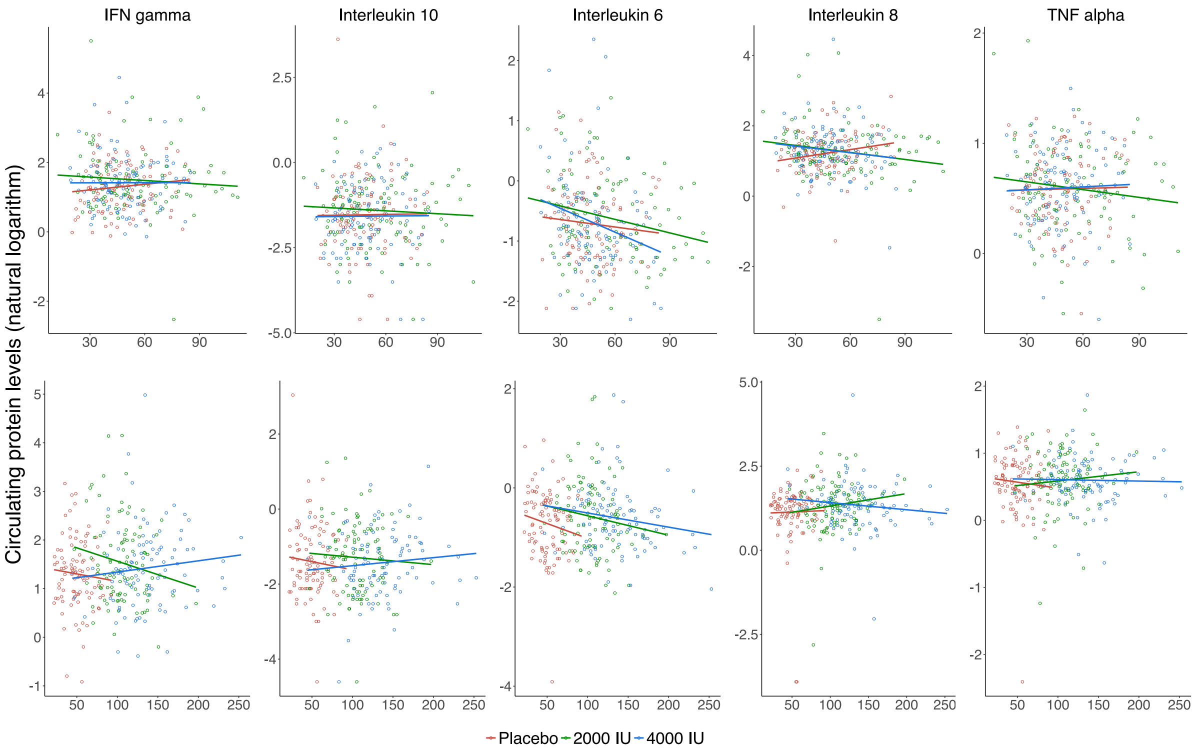

Supplementary Figure 9: Relationship between circulating cytokines and vitamin D supplementation. Scatterplots with regression lines of circulating cytokines at baseline (top) and at 12 months (bottom) by treatment arm (red = placebo, green = 2000 IU, blue = 4000 IU). x-axis = plasma 25(OH)D levels (nmol/L), y-axis = natural logarithm for each corresponding circulating cytokine.

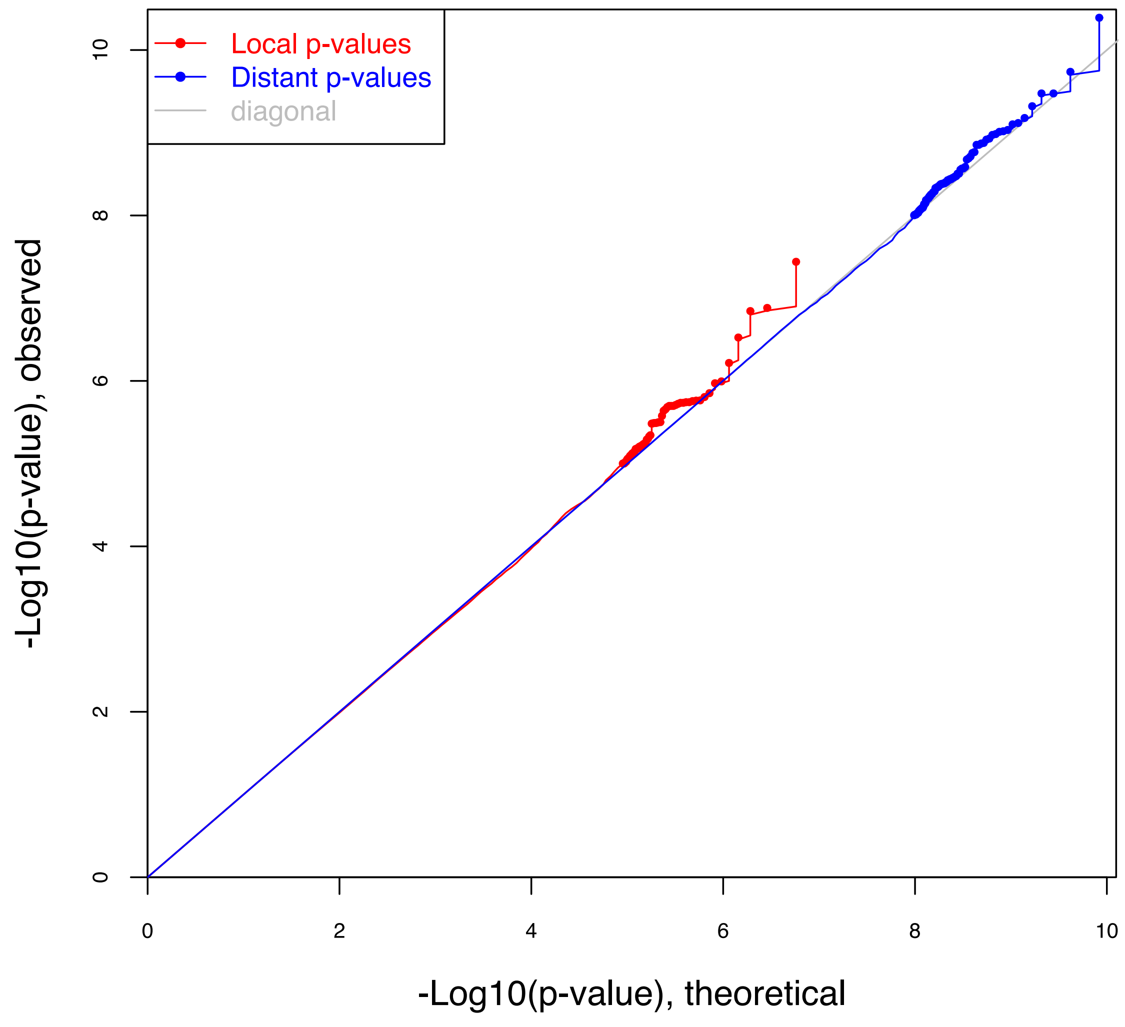

Supplementary Figure 10: Quantile-quantile plot of fold change eQTLs. Expected versus observed p-values of cis (red, local, 5,755,203 p-values) and trans (blue, distant, 8,326,244,157 p-values) eQTLs of fold change gene expression values of vitamin D supplemented individuals (2000IU and 4000IU analysed as one group).
