# Supplementary material for: Genomic Response to Vitamin D Supplementation in the Setting of a Randomized, Placebo-Controlled Trial
Source: eBioMedicine. 2018 Apr 10;31:133–42. doi: 10.1016/j.ebiom.2018.04.010 (PMC6013786; doi:10.1016/j.ebiom.2018.04.010)
Supplement: Supplementary file 8 — Supplementary material 2. [file mmc8.pdf]

Functional genomics of vitamin D supplementation in the BEST-D trial: Data analysis plan

Antonio J Berlanga-Taylor and Julian C Knight

Contact details:

antonio.berlanga@dpag.ox.ac.uk

Computational Genomics Analysis and Training (CGAT), MRC Functional Genomics Unit, Department of Physiology Anatomy & Genetics, University of Oxford, South Parks Road, Oxford OX1 3PT

julian@well.ox.ac.uk

Wellcome Trust Centre for Human Genetics (WTCHG), Nuffield Department of Medicine, University of Oxford, Old Road Campus, Headington, Oxford OX3 7BN

Introduction

This data analysis plan describes the outcomes, comparisons and methods that will be used for the functional genomics analysis of vitamin D supplementation involving the BEST-D clinical trial (www.bestdtrial.org). This is focused on genetic association, gene expression and integrated analysis. These analyses will be carried out by the J Knight group and CGAT in collaboration with the Clinical Trial Service Unit, all at the University of Oxford.

Context of the BEST-D trial

The Biochemical Efficacy and Safety Trial of Vitamin D is a dose-finding trial assessing the biochemical and vascular effects of high dose vitamin D. The rationale, design and protocol are described elsewhere. Below we briefly describe the rationale of the functional genomics analysis of the BEST-D trial.

Functional genomics and molecular analysis in BEST-D

Background

Although animal and experimental models are informative, recent studies have highlighted the difficulty of translating such research findings to humans, particularly in the context of complex diseases (Seok, Warren et al., 2013; Gentile, Nacionales et al., 2014). Higher than expected negative results in clinical trials, exemplified by sepsis (Annane, 2009), have emphasized the need to better understand physiopathological processes and mechanisms of action of drugs.

Randomised clinical trials provide one of the best designs to assess causality and by-pass the difficulties of translating findings from experimental models. Clinical trials are rarely coupled to detailed molecular analysis however and incomplete knowledge of target molecules may cause efficacy trials to result in inconclusive findings. Furthermore, it is known that functional regulatory genetic variation can play a determining role in individual susceptibility to disease (Visscher, Brown et al., 2012) and to drug response (Qiu, Rogers et al., 2014) by modulating RNA expression (Lee, Ye et al., 2014; Fairfax, Humburg et al., 2014). Recently, clinical trial studies have begun using transcriptome comparisons to aid mechanistic understanding and biomarker identification (Beck, Thaci et al., 2014). To our knowledge, no trial has previously studied the impact of genetic variation in response to vitamin D

supplementation and very few studies have assessed the genetics of induced gene expression in clinical trials (Qiu, Rogers et al., 2014).

The aim of functional genomics is to understand how genomes and their products relate to the organism's phenotype. Recent technological and scientific advancements in this field now make it possible to study molecular mechanisms at unparalleled scale. Genome-wide genotyping and gene expression profiling combined with complementary biochemical analysis can be used as global measures of systemic response and mechanism search. These genomic and molecular measures can be associated with phenotypic data to provide an integrated analysis that may yield clinically relevant insights.

Highly interdisciplinary and collaborative efforts are necessary in order to design and execute studies requiring careful sample and data collection for functional genomics analysis in the context of randomised clinical trials. The functional genomic analysis of vitamin D supplementation in BEST-D study is a unique effort to explore how basic science research can work in tandem with large-scale trials. Its aim within the context of the trial is to understand how response to an intervention varies between individuals and treatment groups and to provide insight into the mechanisms of action of vitamin D.

Vitamin D is well known for its classic role in calcium metabolism with deficiency associated with bone disease while there is accumulating evidence pointing to its importance in vascular and immune/inflammatory disorders (Bouillon, Carmeliet et al., 2008; Fraser and Kodicek, 1970; Brumbaugh and Haussler, 1975). Indeed over the last several years it has become clear that vitamin D has multi-system effects and is an important modulator of innate and adaptive immunity with anti-inflammatory actions.

Aim of the functional genomics and molecular analysis in BEST-D study

The overall aim of this study is to investigate the effects of low and high dose vitamin D supplementation on genome-wide gene expression and how this is modulated by genetic variation. To do this, we propose adopting a functional genomics approach to analyse study participants in the BEST-D clinical trial (placebo, low dose or high dose supplementation over a 12-month treatment period).

1. We will measure changes in genome-wide gene expression and blood markers of immunity and inflammation, correlate this with 25(OH)D concentrations and use systems biology approaches to investigate differentially expressed genes, pathways and functional responses.
2. We will investigate the impact of vitamin D supplementation on the functional effects of regulatory genetic variation by mapping gene expression quantitative trait loci (eQTL) in different contexts (placebo, low dose and high dose vitamin D supplementation) and interrogate phenotypic relationships.
3. We will assess the feasibility of carrying out RNA and DNA analysis in the context of a randomised, double-blinded, placebo controlled trial.

Hypothesis of the BEST-D trial and of the molecular effects of vitamin D supplementation

Vitamin D exerts effects in several essential biological processes and perturbations in these may contribute to pathophysiology (Bouillon, Bischoff-Ferrari et al., 2008). Causal evidence is generally lacking but it is possible that vitamin D contributes to the control of vascular calcification, arterial stiffness and/or cardiac hypertrophy

through the regulation of parathyroid hormone (Holick, 2007; Bouillon, Bischoff-Ferrari et al., 2008). It is also proposed that the immune and inflammatory modulating effects of vitamin D may be relevant in both vascular (Wong, Leisegang et al., 2014) and non-vascular disease (Donate-Correa, Dominguez-Pimentel et al., 2014; Busse, Bale et al., 2013).

In the context of inflammation, we hypothesise that vitamin D produces beneficial effects by reducing chronic inflammation and this can be measured in blood cells through molecular biomarkers. Following vitamin D supplementation, we anticipate a shift in the transcriptomic profile of participants, in particular in relation to pro- and anti-inflammatory molecules and other associated molecules.

Previous studies have investigated the relationship of vitamin D to gene expression (Carlberg, 2014; Hossein-nezhad, Spira et al., 2013). These have generally been in experimental models, cross-sectional studies or in studies with small sample sizes. The present investigation will use a randomised trial design, the BEST-D trial, to relate changes in plasma 25(OH)D to changes in gene expression in healthy older people as well as to genetic variation, biochemical measures and phenotypic information.

Design

The BEST-D design is a double blind, randomised, placebo controlled trial with before and after and group comparisons for supplementation with 2000 IU and 4000 IU of vitamin D with 100 participants in each group. The primary efficacy assessment is of intention to treat. As part of the BEST-D trial, samples for DNA, RNA and plasma measurements were obtained from participants together with physiologic, phenotypic and safety measures as described separately in the BEST-D trial protocol. Samples for DNA and RNA analyses will be obtained at baseline (0) and 12 months.

Outcomes

a. Primary outcomes

- Genome-wide differential gene expression for peripheral blood leukocytes following vitamin D supplementation (profiling at baseline and 12 months, resolution of differentially expressed genes);
- Genome-wide single nucleotide (SNP) genotyping of study cohort (baseline);
- eQTL mapping of local (likely cis-acting) and distant (trans-acting) SNP markers associated with gene expression in different contexts of vitamin D supplementation.

b. Secondary outcomes

- Pathway and systems biology analysis of differentially expressed genes associated with vitamin D supplementation and co-expression gene modules;
- Analysis of genotype-transcriptome-plasma 25(OH)D associations;
- Analysis of quantitative trait loci (QTL) for plasma levels of 25(OH)D in BEST-D participants using genetic variants previously associated by genome-wide association studies (Wang, Zhang et al., 2010; Ahn, Yu et al., 2010);
- Analysis of molecular associations between genotype, gene expression, plasma 25(OH)D and pre-specified plasma measurements (PTH, calcium, albumin, phosphate, creatinine, alkaline phosphatase, total cholesterol, LDL-C, HDL-C, triglycerides, apolipoprotein A, apolipoprotein B, hsCRP and NT-proBNP). These will be primarily carried out for concurrent measurements (i.e. for those at

baseline and 12 months). See also the protocol and data analysis plan for BEST-D;

- Analysis of the association between molecular measurements and pre-specified phenotype measurements including fractures, respiratory infections, body mass index, bone density measurements, glomerular filtration rate and urinary albumin/creatinine ratio;
- Measurement of pro- and anti-inflammatory cytokines prioritised by the mRNA profiling described in the primary outcome.

c. Tertiary outcomes

- Quantitative trait locus (QTL) analysis of pre-specified plasma measurements for those mentioned above;
- Analysis of association of molecular and physiologic measures including systolic and diastolic blood pressure, heart rate, and arterial stiffness measured at 12 months;
- Analysis of association of molecular and echocardiographic measures for a pre-specified sub-group at 12 months.

Comparisons

a. Primary comparisons of the primary outcomes:

- All participants allocated 100 µg (4,000 IU) vitamin D_3 daily (data collected at 12 months) vs. baseline;
- All participants allocated 100 µg (4,000 IU) vitamin D_3 daily vs. all participants allocated placebo.
- All participants allocated 100 µg (4,000 IU) vitamin D_3 daily vs. all participants allocated 50 µg (2,000 IU) vitamin D_3 daily;
- All participants allocated 50 µg (2,000 IU) vitamin D_3 daily vs. baseline
- All participants allocated 50 µg (2,000 IU) vitamin D_3 daily vs. participants allocated placebo.

b. Secondary comparisons of the primary outcomes:

- Participants allocated any active vitamin D_3 dose (50 µg or 100 µg daily) versus participants allocated placebo.

c. Tertiary comparison of the primary outcomes:

All participants (4,000 IU, 2,000 IU and placebo) will be analysed according to treatment allocation separately using the following covariates (baseline characteristics):

- Sex
- Age (<70, ≥70 years)
- BMI (<25, ≥25 to <30, ≥30 kg/m^2)
- Plasma 25(OH)D concentrations (<25, ≥25 to <50, ≥50 nmol/L)
- Dietary calcium intake (<800, ≥800 to <1000, ≥1000 mg/day)
- Estimated glomerular filtration rate (eGFR) (<60, ≥60 to <90, ≥90 ml/min/1.73 m^2)
- Prior history of cardiovascular disease
- Prior history of cancer

Although intervals have been assigned for some variables, continuous data will be used where appropriate. A generalized linear model for gene expression and

baseline characteristics will be used as part of this analysis as described in similar studies (Smyth, 2004) and specified below.

d. Comparisons of secondary and tertiary outcomes will include:
- Changes from baseline within each treatment allocation;
- Participants allocated 100 µg vitamin D_3 daily vs. participants allocated placebo;
- Participants allocated 50 µg vitamin D_3 daily vs. participants allocated placebo;
- Participants allocated any active vitamin D_3 dose (50 µg or 100 µg daily) vs. participants allocated placebo;
- In some instances, comparisons will be made between vitamin D_3 dose groups to understand dose responses.

<u>Details of analyses</u>

a. Analysis methods overview

Our analysis follows the BEST-D data analysis plan and will carry out comparisons as intention-to-treat analyses. The main analyses at each stage are outlined below. Methods are intricate and evolving however and several stages are dependent on the particular characteristics and quality of the acquired measurements. Here we only provide a brief overview of the general steps that will be taken at each stage.

b. Data collection and management

All results will be handled according to the trial and institutional guidelines in secure servers within the University of Oxford in accordance with current regulations.

c. Data pre-processing, quality control and statistical analysis

Genome-wide genomic measurements involve hundreds of thousands of data points and typically require pre-processing and quality control (QC) analysis. These will be carried out according to published methods and current best practice. A brief description, including software to use in some instances, is provided below as guidance and to exemplify the planned approaches.

DNA extraction and genotyping

Genome-wide genotyping and statistical methods of genetic association have become widely available, particularly over the last five years, and have proved highly reproducible (Visscher, Brown et al., 2012). We will adhere to current recommendations and best practice. The following general steps are required and are described in more detail elsewhere:

1) DNA extraction and genotyping as specified separately;
2) Quality control of subject and SNP genotyping data includes:
   a. Gender misidentification;
   b. Subject relatedness, duplication and divergent ancestry;
   c. Individuals with elevated missing data rates or outlying heterozygosity rate;
   d. Identification of markers (SNPs) with excessive missing data rates;
   e. Identification of differing genotype call rates between groups;
   f. SNP quality (filtering of monomorphic SNPs, SNPs with missing values or nonsense values; imputation quality; low call rate; violation of Hardy-Weinberg Equilibrium; duplication; and minimum allele frequency);
   g. Association tests will be carried out as described previously (Sham and Purcell, 2014; Pirinen, Donnelly et al., 2013) using frequentist methods

(typically based on Chi-square tests) and allowing for multiple testing correction. We will consider Bayesian methods for specific instances, such as fine mapping, as previously described (Stahl, Wegmann et al., 2012; Stephens and Balding, 2009). Association tests will be carried out at the appropriate step for the particular analysis (e.g. during QTL analyses).

Imputation of genotype data

Genotype imputation is a computational method that can increase the power of genetic association studies (Marchini and Howie, 2010). It has been used commonly in genome-wide association studies to infer missing genotypes, fine-map associations and harmonise data sets (Porcu, Sanna et al., 2013). We plan to perform imputation and use data to fine-map significant associations. The following steps and tools are generally necessary during imputation:

1) Harmonisation of markers, reference panel and pre-imputation quality control;
2) Phasing of genotypes, imputation, post-imputation quality control.

Microarray gene expression profiling

Genome-wide gene expression profiling has become a common approach to interrogate cellular function and provide mechanistic insight. Microarray methods are available and in general statistical methods are mature and well accepted. Established protocols, statistical approaches and software are available for analysis of transcriptomic data that can also account for multifactor designed experiments. Simultaneous comparisons between many RNA targets with a variety of experimental conditions and predictors can be performed. Analytical workflows are generally intricate however and require normalisation; outlier analysis; adjustment for confounding effects; differential expression analysis, and integration with genotype, phenotype, external sources (e.g. gene annotations) or other available data. Analysis of differential expression generally relies on the use of linear models (Smyth, 2004) although Bayesian approaches can also be applied. We will include the following steps in our data analysis:

1) RNA extraction, cDNA conversion and microarray measurements as specified separately;
2) Normalization of microarray data, adjustment for confounding effects and differential expression analysis:
    a. Quality assessment;
    b. Pre-processing and probe filtering;
    c. Control of possible confounding effects;
    d. Differential expression analysis, uniquely expressed genes and downstream analysis.

Expression quantitative trait loci (eQTL) analysis

Several statistical models have been described for eQTL analysis (Battle and Montgomery, 2014; Montgomery and Dermitzakis, 2009). We plan to follow our published approaches (Fairfax 2012, 2014) including using an additive linear model as implemented in the R package MatrixEQTL (Shabalin, 2012) with inclusion as appropriate of covariates including dominant principal components of variance and comparing its reproducibility to other approaches such as an ANOVA model (Shabalin, 2012). Although our study is based on a randomised trial, we can

compare eQTL results for reproducibility while accounting for the effects of hidden factors (Gao, Tignor et al., 2014; Fusi, Stegle et al., 2012). We will investigate context-specificity for vitamin D supplementation using multivariate Bayesian (Petretto, Bottolo et al., 2010; Imholte, Scott-Boyer et al., 2013) and GWAS meta-analysis (hierarchical model) (Flutre, Wen et al., 2013) approaches. Once genotype and gene expression data have been processed as described above we will perform the eQTL analysis as mentioned including the following steps:

1) Probe filtering;
2) Confounding factors analysis;
3) Definition and identification of *cis* and *trans* SNPs;
4) Conditional analysis for fine mapping;
5) Quantification statistics.

Functional annotation of eQTL signals and overlap with known associations

Once we have a list of eQTL signals we will search public databases for other known associations and overlap with disease-associated variants to aid biological interpretation. The main studies and databases that we expect to use include ENCODE (Dunham, Kundaje et al., 2012), FANTOM5 (Andersson, Gebhard et al., 2014; Forrest, Kawaji et al., 2014), GTex (GTex Consortium, 2013), Epigenome roadmap (Bernstein, Stamatoyannopoulos et al., 2010), GWAS catalogue (Hindorff, Sethupathy et al., 2009), GWAS central (Beck, Hastings et al., 2014), amongst others. In the case of overlap with disease-variants we will use GWAS disease categories as described (Fairfax, Humburg et al., 2014). Enrichment of eQTLs in GWAS categories will be tested, for example using Fisher's exact test. Further enrichment analysis will also be carried out using GAT if appropriate (Heger, Webber et al., 2013). Background values will be those appropriate to the test (i.e. all SNPs) as will the comparison against expected values (i.e. all significant *cis*-SNPs). We will consider GWAS SNPs to overlap with significant eQTL SNPs if these are in high linkage disequilibrium (r^2 >0.8).

Pathway, network and co-expression analyses

Pathway analyses will be carried out with one or several packages, namely Ingenuity Pathway Analysis (IPA, Ingenuity Systems), in order to define gene networks, upstream regulators, and canonical pathways. Other database repositories and analysis tools such as DAVID (Huang da, Sherman et al., 2009) and GREAT (McLean, Bristor et al., 2010) will be used for comparison if necessary. We will perform gene co-expression network analysis using WGCNA (Langfelder and Horvath, 2008). If particular gene signatures are identified, we will make further comparisons against specific repositories. For example, if an interferon regulated pathway were identified, we will used published articles and databases such as: http://interferome.its.monash.edu.au/interferome/home.jspx (Rusinova, Forster et al., 2013). Significance thresholds will remain as those stated below after p-value adjustment where required (i.e. 1%, 5% and 10% values for interpretation, sensitivity and comparison with 5% as the accepted minimum threshold).

Methods for handling missing data

Pairwise deletion or multiple imputation of missing data as deemed appropriate for the particular analysis and variable will be used. For multiple imputation, results will be compared with those from complete-case analyses.

Allowance for multiplicity of comparisons

We will use approaches based on false discovery rates and multiple-testing correction (such as Bonferroni and Benjamini-Hochberg methods), taking into account the type of measure and evidence from other studies as found relevant. We will aim to carry out permutation tests where appropriate.

Tests for heterogeneity effects

For the main effects and comparisons (i.e. gene expression) we will carry out comparisons between sub-groups of participants to tests for heterogeneity with allowance for multiple comparisons and for other differences between the subgroups. We will use this to determine whether the effects in these subgroups are significantly different from the overall effect. Given the availability of genetic variation data and the study objective of identifying functional variants, the differences that we may observe could be due to a true biological effect as previously reported in other contexts (Lee, Ye et al., 2014; Fairfax, Humburg et al., 2014; Qiu, Rogers et al., 2014; Visscher, Brown et al., 2012).

Use of Bayesian and non-standard statistical techniques

Current approaches in computational and statistical genomics largely apply frequentist methods. Although some of the analytical methods are mature, such as those for genetic association and gene expression, it remains a rapidly evolving field. We may compare methods and approaches where possible and appropriate to help determine sensitivity and reproducibility of findings.

Covariates

Sex, age, body mass index, dietary calcium intake, prior history of disease (cardiovascular disease, cancer); total and differential blood cell count.

Possible batch and hidden effects, particularly in genotype and gene expression data, will be resolved using principal components analysis and accounted for. In gene expression analysis this is standard and sample randomisation for microarray measurements will be performed to minimise this.

Pre-specified sub-group analysis

- Individuals with particular genetic variants may show a different response to vitamin D_3 supplementation. If these individuals are identified by eQTL mapping, we will carry out further analyses to try and gain further understanding of the mechanisms and possible consequences.
- We will carry out an analysis of molecular associations with echocardiographic data for the sub-group of individuals for whom these will be available.
- We will analyse separately individuals who present any adverse outcome.

Post-hoc analysis

Functional genomics approaches typically make use of genome-wide strategies that assay many functions and relationships. The outcomes and comparisons described above may generate hypothesis that will require additional experimentation and analysis. They will also likely require validation and/or follow-up work. These analyses and further experiments cannot be specified *a priori* and may for example involve the measurement of other molecules, epigenetic marks, integration with other

molecular or phenotypic data, comparison with other cohorts and/or integration with other experiment models or other approaches as deemed appropriate.

Practicalities relating to the blinding of the trial statistician

We will adhere to established blinding protocols. All raw data (gene expression and genotyping) will be generated blind to treatment allocation and a complete copy of this data provided to the BEST-D trialists before commencing data processing, quality control and analysis. Functional genomic data will be generated and analysed outside CTSU as specialist facilities, high performance computing facilities, knowledge and expertise are required.

Documentation and reproducibility of analyses

Computational pipelines will be made available and are expected to be automated, transparent and reproducible. Computer environment, available software and other dependencies may need to be set-up before pipelines can be run successfully in other systems however.

References

Ahn, J., Yu, K., Stolzenberg-Solomon, R., et al. (2010). "Genome-wide association study of circulating vitamin D levels." Hum Mol Genet **19**(13): 2739-2745.

Andersson, R., Gebhard, C., Miguel-Escalada, I., et al. (2014). "An atlas of active enhancers across human cell types and tissues." Nature **507**(7493): 455-461.

Annane, D. (2009). "Improving clinical trials in the critically ill: unique challenge--sepsis." Crit Care Med **37**(1 Suppl): S117-128.

Battle, A. and Montgomery, S. B. (2014). "Determining causality and consequence of expression quantitative trait loci." Hum Genet **133**(6): 727-735.

Beck, L. A., Thaci, D., Hamilton, J. D., et al. (2014). "Dupilumab treatment in adults with moderate-to-severe atopic dermatitis." N Engl J Med **371**(2): 130-139.

Beck, T., Hastings, R. K., Gollapudi, S., Free, R. C. and Brookes, A. J. (2014). "GWAS Central: a comprehensive resource for the comparison and interrogation of genome-wide association studies." Eur J Hum Genet **22**(7): 949-952.

Bernstein, B. E., Stamatoyannopoulos, J. A., Costello, J. F., et al. (2010). "The NIH Roadmap Epigenomics Mapping Consortium." Nat Biotechnol **28**(10): 1045-1048.

Bouillon, R., Bischoff-Ferrari, H. and Willett, W. (2008). "Vitamin D and health: perspectives from mice and man." J Bone Miner Res **23**(7): 974-979.

Bouillon, R., Carmeliet, G., Verlinden, L., et al. (2008). "Vitamin D and human health: lessons from vitamin D receptor null mice." Endocr Rev **29**(6): 726-776.

Brumbaugh, P. F. and Haussler, M. R. (1975). "Specific binding of 1alpha,25-dihydroxycholecalciferol to nuclear components of chick intestine." J Biol Chem **250**(163254): 1588-1594.

Busse, B., Bale, H. A., Zimmermann, E. A., et al. (2013). "Vitamin D deficiency induces early signs of aging in human bone, increasing the risk of fracture." Sci Transl Med **5**(193): 193ra188.

Carlberg, C. (2014). "Genome-wide (over)view on the actions of vitamin D." Front Physiol **5**: 167.

Donate-Correa, J., Dominguez-Pimentel, V., Muros-de-Fuentes, M., et al. (2014). "Beneficial effects of selective vitamin D receptor activation by paricalcitol in chronic kidney disease." Curr Drug Targets **15**(7): 703-709.

Dunham, I., Kundaje, A., Aldred, S. F., et al. (2012). "An integrated encyclopedia of DNA elements in the human genome." Nature **489**(7414): 57-74.

Fairfax, B. P., Humburg, P., Makino, S., et al. (2014). "Innate immune activity conditions the effect of regulatory variants upon monocyte gene expression." Science **343**(6175): 1246949.

Flutre, T., Wen, X., Pritchard, J. and Stephens, M. (2013). "A statistical framework for joint eQTL analysis in multiple tissues." PLoS Genet **9**(5): e1003486.

Forrest, A. R., Kawaji, H., Rehli, M., et al. (2014). "A promoter-level mammalian expression atlas." Nature **507**(7493): 462-470.

Fraser, D. R. and Kodicek, E. (1970). "Unique biosynthesis by kidney of a biological active vitamin D metabolite." Nature **228**(4319631): 764-766.

Fusi, N., Stegle, O. and Lawrence, N. D. (2012). "Joint modelling of confounding factors and prominent genetic regulators provides increased accuracy in genetical genomics studies." PLoS Comput Biol **8**(1): e1002330.

Gao, C., Tignor, N. L., Salit, J., et al. (2014). "HEFT: eQTL analysis of many thousands of expressed genes while simultaneously controlling for hidden factors." Bioinformatics **30**(3): 369-376.

Gentile, L. F., Nacionales, D. C., Lopez, M. C., et al. (2014). "A better understanding of why murine models of trauma do not recapitulate the human syndrome." Crit Care Med **42**(6): 1406-1413.

GTex Consortium (2013). "The Genotype-Tissue Expression (GTEx) project." Nat Genet **45**(6): 580-585.

Heger, A., Webber, C., Goodson, M., Ponting, C. P. and Lunter, G. (2013). "GAT: a simulation framework for testing the association of genomic intervals." Bioinformatics.

Hindorff, L. A., Sethupathy, P., Junkins, H. A., et al. (2009). "Potential etiologic and functional implications of genome-wide association loci for human diseases and traits." Proc Natl Acad Sci U S A **106**(23): 9362-9367.

Holick, M. F. (2007). "Vitamin D deficiency." N Engl J Med **357**(3): 266-281.

Hossein-nezhad, A., Spira, A. and Holick, M. F. (2013). "Influence of vitamin D status and vitamin D3 supplementation on genome wide expression of white blood cells: a randomized double-blind clinical trial." PLoS One **8**(3): e58725.

Huang da, W., Sherman, B. T. and Lempicki, R. A. (2009). "Systematic and integrative analysis of large gene lists using DAVID bioinformatics resources." Nat Protoc **4**(1): 44-57.

Imholte, G. C., Scott-Boyer, M. P., Labbe, A., Deschepper, C. F. and Gottardo, R. (2013). "iBMQ: a R/Bioconductor package for integrated Bayesian modeling of eQTL data." Bioinformatics **29**(21): 2797-2798.

Langfelder, P. and Horvath, S. (2008). "WGCNA: an R package for weighted correlation network analysis." BMC Bioinformatics **9**: 559.

Lee, M. N., Ye, C., Villani, A. C., et al. (2014). "Common genetic variants modulate pathogen-sensing responses in human dendritic cells." Science **343**(6175): 1246980.

Marchini, J. and Howie, B. (2010). "Genotype imputation for genome-wide association studies." Nat Rev Genet **11**(7): 499-511.

McLean, C. Y., Bristor, D., Hiller, M., et al. (2010). "GREAT improves functional interpretation of cis-regulatory regions." Nat Biotechnol **28**(5): 495-501.

Montgomery, S. B. and Dermitzakis, E. T. (2009). "The resolution of the genetics of gene expression." Hum Mol Genet **18**(R2): R211-215.

Petretto, E., Bottolo, L., Langley, S. R., et al. (2010). "New insights into the genetic control of gene expression using a Bayesian multi-tissue approach." PLoS Comput Biol **6**(4): e1000737.

Pirinen, M., Donnelly, P. and Spencer, C. C. (2013). "Efficient computation with a linear mixed model on large-scale data sets with applications to genetic studies." The Annals of Applied Statistics **7**(1): 369-390.

Porcu, E., Sanna, S., Fuchsberger, C. and Fritsche, L. G. (2013). "Genotype imputation in genome-wide association studies." Curr Protoc Hum Genet **Chapter 1**: Unit 1 25.

Qiu, W., Rogers, A. J., Damask, A., et al. (2014). "Pharmacogenomics: Novel Loci Identification via Integrating Gene Differential Analysis and eQTL Analysis." Hum Mol Genet.

Rusinova, I., Forster, S., Yu, S., et al. (2013). "Interferome v2.0: an updated database of annotated interferon-regulated genes." Nucleic Acids Res **41**(Database issue): D1040-1046.

Seok, J., Warren, H. S., Cuenca, A. G., et al. (2013). "Genomic responses in mouse models poorly mimic human inflammatory diseases." Proc Natl Acad Sci U S A **110**(9): 3507-3512.

Shabalin, A. A. (2012). "Matrix eQTL: ultra fast eQTL analysis via large matrix operations." Bioinformatics **28**(10): 1353-1358.

Sham, P. C. and Purcell, S. M. (2014). "Statistical power and significance testing in large-scale genetic studies." Nat Rev Genet **15**(5): 335-346.

Smyth, G. K. (2004). "Linear models and empirical bayes methods for assessing differential expression in microarray experiments." Stat Appl Genet Mol Biol **3**: Article3.

Stahl, E. A., Wegmann, D., Trynka, G., et al. (2012). "Bayesian inference analyses of the polygenic architecture of rheumatoid arthritis." Nat Genet **44**(5): 483-489.

Stephens, M. and Balding, D. J. (2009). "Bayesian statistical methods for genetic association studies." Nat Rev Genet **10**(10): 681-690.

Visscher, P. M., Brown, M. A., McCarthy, M. I. and Yang, J. (2012). "Five years of GWAS discovery." Am J Hum Genet **90**(1): 7-24.

Wang, T. J., Zhang, F., Richards, J. B., et al. (2010). "Common genetic determinants of vitamin D insufficiency: a genome-wide association study." Lancet **376**(9736): 180-188.

Wong, M. S., Leisegang, M. S., Kruse, C., et al. (2014). "Vitamin D Promotes Vascular Regeneration." Circulation.
