# Supplementary material for: Genomic Response to Vitamin D Supplementation in the Setting of a Randomized, Placebo-Controlled Trial
Source: eBioMedicine. 2018 Apr 10;31:133–42. doi: 10.1016/j.ebiom.2018.04.010 (PMC6013786; doi:10.1016/j.ebiom.2018.04.010)
Supplement: Supplementary file 9 — Supplementary material 3. [file mmc9.pdf]

Lay summary and wider context

Effect of vitamin D supplementation on biomarkers of inflammation and immune function: functional genomics analysis of the BEST-D trial

Antonio J. Berlanga-Taylor, Katherine Plant, Andrew Dahl, Evelyn Lau, Michael Hill, David Sims, Andreas Heger, Jonathan Emberson, Jane Armitage, Robert Clarke and Julian C. Knight

Review of current evidence

The rapid growth in genomics methods have allowed wider applicability and can be used as powerful tools in clinical trials. Several fields have increasingly adopted the use of high-throughput molecular technologies to help address important biomedical questions. Dozens of studies have shown disease associations with vitamin D deficiency. Strong evidence of causal relationships is generally lacking however and several trials have been performed or are under way to address these issues. There continues to be discussion as to what the adequate dose of vitamin D should be however.

In-vitro and *ex-vivo* studies of gene expression and vitamin D receptor binding to DNA have shown that vitamin D has genome-wide effects (1-3). The number of genes and effect sizes vary considerably between studies and may be due to a number of reasons including cell-type, genetic variability, and posology. Studies have generally used 1,25 dihydroxyvitamin D at supra-physiological doses for short periods (hours to days). Before finalising the design of our study (December 2011), we did not identify clinical trials which had measured genome-wide gene expression following vitamin D supplementation (EU Clinical Trials Register and ClinicalTrials.gov; some studies measured candidate genes). In the Gene Expression Omnibus repository, for example, we found 42 datasets when searching for “clinical trial” in expression profiling by array or high throughput sequencing. Of these, the majority (25) related to cancer

and none to vitamin D. Two previous studies did not find an effect between serum levels of 25(OH)D and a small number of cytokines after vitamin D supplementation (4, 5).

More recent findings have had mixed results depending on the study population and cellular or molecular trait measured amongst many other variables. A small open-label trial showed changes in immune cellular composition following cholecalciferol in patients with systemic lupus erythematosus (6). A trial in 131 women with vitamin D deficiency did not find changes in a candidate set of immune genes (7). Another clinical trial reported colonic gene expression changes at 4-weeks following 1,25-dihydroxyvitamin D_3 (8). Other studies using candidate approaches for transcriptomic or epigenetic marks correlated to levels of 25(OH)D found short and mid-term changes (9-11). A daily 5000 IU vitamin D_3 supplement for 14 weeks in 39 active men increased cathelicidin and immunoglobulin A compared to placebo but did not test transcriptomic changes (12). A trial in a small number of individuals (n=8) showed that 291 genes were differentially expressed following either 400 IU or 2000 IU vitamin D_3 supplementation at 2 months compared to baseline in white blood cells (13). This study did not find differences when looking at either dose alone and did not include a placebo control however. A recent placebo controlled trial with a sample size of 47 individuals per arm with reduced glucose tolerance did not find genome wide gene expression differences following vitamin D supplementation (14). Other work has observed differences in CD4+ T cell frequencies (15, 16), short term changes in candidate genes (17), differences in response to treatment based on genotype (18) or baseline vitamin D circulating levels (19). The study by (19) did not detect differences in candidate gene expression however.

Research appears limited in terms of investigating the genetic effects on vitamin D supplementation or dietary intake. A recent study investigated interaction between a low-

frequency genetic variant with vitamin D dietary intake (mostly from food frequency questionnaires) in >9,000 individuals but did not find significant differences (20).

Added value and implications for future work

To our knowledge, this trial is one of the largest of its kind and addresses important limitations of previous studies in terms of sample size, design and duration. Importantly, few or no studies have had placebo controls when assessing gene expression following vitamin D supplementation and have not accounted for the confounding effect of time. Using a gold standard design and sufficient power we are unable to observe transcriptomic or circulating cytokine differences in whole blood from older aged community dwelling individuals nor were we able to detect whether genetic variation affects gene expression following vitamin D supplementation. Effects of genetic changes of this type are often called context specific quantitative trait loci (QTL), which in this case would represent drug response QTLs. Context-specific QTLs can reveal detailed information on molecular mechanisms.

Vitamin D undoubtedly has wide ranging effects, largely mediated by transcriptional changes. These effects may vary according to individual and environmental circumstances, genetic make-up, cell type measured, dose, season, amongst many other factors. Genetic and physiological mechanisms regulating the effects of vitamin D are complex and likely robust to short and mid-term environmental changes. Indeed, regions near vitamin D related genes appear to be under selective pressure (2, 21, 22) but vitamin D metabolism genes do not seem intolerant to genetic mutations or to copy number variation (23, 24). Dozens of observational studies have shown a relationship between 25(OH)D deficiency and higher risk of death or disease. Meta-analyses of randomised controlled trials of vitamin D supplementation have found small but statistically significant decreases in all-cause mortality (25). A survey of the literature showed that 24 vitamin D Mendelian Randomization studies have been undertaken,

indicating the intense interest on the causal relationships between circulating 25(OH)D and disease (Supplementary Table 5). Similarly, many trials are now being planned or are under way to test whether vitamin D supplementation can treat or prevent some of these conditions. In the 12 months to June 12, 2017 for example, 52 clinical trials using vitamin D or an analogue have been published (Supplementary Table 6). However, few or none have systematically investigated possible mechanisms and heterogeneity in drug response. Our results can guide future research and aid in the design of further studies elucidating the mechanisms of action of vitamin D.

References

1. Wang TT, Tavera-Mendoza LE, Laperriere D, Libby E, MacLeod NB, Nagai Y, et al. Large-scale in silico and microarray-based identification of direct 1,25-dihydroxyvitamin D3 target genes. Mol Endocrinol. 2005;19(11):2685-95.
2. Ramagopalan SV, Heger A, Berlanga AJ, Maugeri NJ, Lincoln MR, Burrell A, et al. A ChIP-seq defined genome-wide map of vitamin D receptor binding: associations with disease and evolution. Genome Res. 2010;20(10):1352-60.
3. Seuter S, Neme A, Carlberg C. Epigenome-wide effects of vitamin D and their impact on the transcriptome of human monocytes involve CTCF. Nucleic Acids Res. 2016;44(9):4090-104.
4. Jorde R, Sneve M, Torjesen PA, Figenschau Y, Goransson LG, Omdal R. No effect of supplementation with cholecalciferol on cytokines and markers of inflammation in overweight and obese subjects. Cytokine. 2010;50(2):175-80.
5. Yusupov E, Li-Ng M, Pollack S, Yeh JK, Mikhail M, Aloia JF. Vitamin d and serum cytokines in a randomized clinical trial. International journal of endocrinology. 2010;2010.
6. Terrier B, Derian N, Schoindre Y, Chaara W, Geri G, Zahr N, et al. Restoration of regulatory and effector T cell balance and B cell homeostasis in systemic lupus erythematosus patients through vitamin D supplementation. Arthritis research & therapy. 2012;14(5):R221.
7. Das M, Tomar N, Sreenivas V, Gupta N, Goswami R. Effect of vitamin D supplementation on cathelicidin, IFN-gamma, IL-4 and Th1/Th2 transcription factors in young healthy females. Eur J Clin Nutr. 2014;68(3):338-43.
8. Protiva P, Pendyala S, Nelson C, Augenlicht LH, Lipkin M, Holt PR. Calcium and 1,25-dihydroxyvitamin D3 modulate genes of immune and inflammatory pathways in the human colon: a human crossover trial. Am J Clin Nutr. 2016;103(5):1224-31.
9. Seuter S, Virtanen JK, Nurmi T, Pihlajamaki J, Mursu J, Voutilainen S, et al. Molecular evaluation of vitamin D responsiveness of healthy young adults. J Steroid Biochem Mol Biol. 2016.
10. Carlberg C, Seuter S, de Mello VD, Schwab U, Voutilainen S, Pulkki K, et al. Primary vitamin D target genes allow a categorization of possible benefits of vitamin D(3) supplementation. PLoS One. 2013;8(7):e71042.
11. Saksa N, Neme A, Ryynanen J, Uusitupa M, de Mello VD, Voutilainen S, et al. Dissecting high from low responders in a vitamin D3 intervention study. J Steroid Biochem Mol Biol. 2015;148:275-82.
12. He CS, Fraser WD, Tang J, Brown K, Renwick S, Rudland-Thomas J, et al. The effect of 14 weeks of vitamin D3 supplementation on antimicrobial peptides and proteins in athletes. Journal of sports sciences. 2016;34(1):67-74.
13. Hossein-nezhad A, Spira A, Holick MF. Influence of vitamin D status and vitamin D3 supplementation on genome wide expression of white blood cells: a randomized double-blind clinical trial. PLoS One. 2013;8(3):e58725.
14. Pasing Y, Fenton CG, Jorde R, Paulssen RH. Changes in the human transcriptome upon vitamin D supplementation. J Steroid Biochem Mol Biol. 2017;173:93-9.
15. Smolders J, Menheere P, Thewissen M, Peelen E, Tervaert JWC, Hupperts R, et al. Regulatory T cell function correlates with serum 25-hydroxyvitamin D, but not with 1,25-dihydroxyvitamin D, parathyroid hormone and calcium levels in patients with relapsing remitting multiple sclerosis. J Steroid Biochem Mol Biol. 2010;121(20211254):243-6.
16. Smolders J, Peelen E, Thewissen M, Cohen Tervaert JW, Menheere P, Hupperts R, et al. Safety and T cell modulating effects of high dose vitamin D3 supplementation in multiple sclerosis. PLoS One. 2010;5(12):e15235.

17. Vukic M, Neme A, Seuter S, Saksa N, de Mello VD, Nurmi T, et al. Relevance of vitamin D receptor target genes for monitoring the vitamin D responsiveness of primary human cells. PLoS One. 2015;10(4):e0124339.
18. Martineau AR, Timms PM, Bothamley GH, Hanifa Y, Islam K, Claxton AP, et al. High-dose vitamin D(3) during intensive-phase antimicrobial treatment of pulmonary tuberculosis: a double-blind randomised controlled trial. Lancet. 2011;377(9761):242-50.
19. Laursen JH, Sondergaard HB, Sorensen PS, Sellebjerg F, Oturai AB. Vitamin D supplementation reduces relapse rate in relapsing-remitting multiple sclerosis patients treated with natalizumab. Multiple sclerosis and related disorders. 2016;10:169-73.
20. Manousaki D, Dudding T, Haworth S, Hsu YH, Liu CT, Medina-Gomez C, et al. Low-Frequency Synonymous Coding Variation in CYP2R1 Has Large Effects on Vitamin D Levels and Risk of Multiple Sclerosis. Am J Hum Genet. 2017;101(2):227-38.
21. Mathieson I, Lazaridis I, Rohland N, Mallick S, Patterson N, Roodenberg SA, et al. Genome-wide patterns of selection in 230 ancient Eurasians. Nature. 2015;528(7583):499-503.
22. Beleza S, Santos AM, McEvoy B, Alves I, Martinho C, Cameron E, et al. The timing of pigmentation lightening in Europeans. Molecular biology and evolution. 2013;30(1):24-35.
23. Lek M, Karczewski KJ, Minikel EV, Samocha KE, Banks E, Fennell T, et al. Analysis of protein-coding genetic variation in 60,706 humans. Nature. 2016;536(7616):285-91.
24. Ruderfer DM, Hamamsy T, Lek M, Karczewski KJ, Kavanagh D, Samocha KE, et al. Patterns of genic intolerance of rare copy number variation in 59,898 human exomes. Nat Genet. 2016.
25. Bjelakovic G, Gluud LL, Nikolova D, Whitfield K, Wetterslev J, Simonetti RG, et al. Vitamin D supplementation for prevention of mortality in adults. Cochrane Database Syst Rev. 2014;1:CD007470.
